# Supplementary figures and images for: Opposing action of the FLR-2 glycoprotein hormone and DRL-1/FLR-4 MAP kinases balance p38-mediated growth and lipid homeostasis in C. elegans
Source: PLoS Biol. 2023 Sep 29;21(9):e3002320. doi: 10.1371/journal.pbio.3002320 (PMC10566725; doi:10.1371/journal.pbio.3002320)

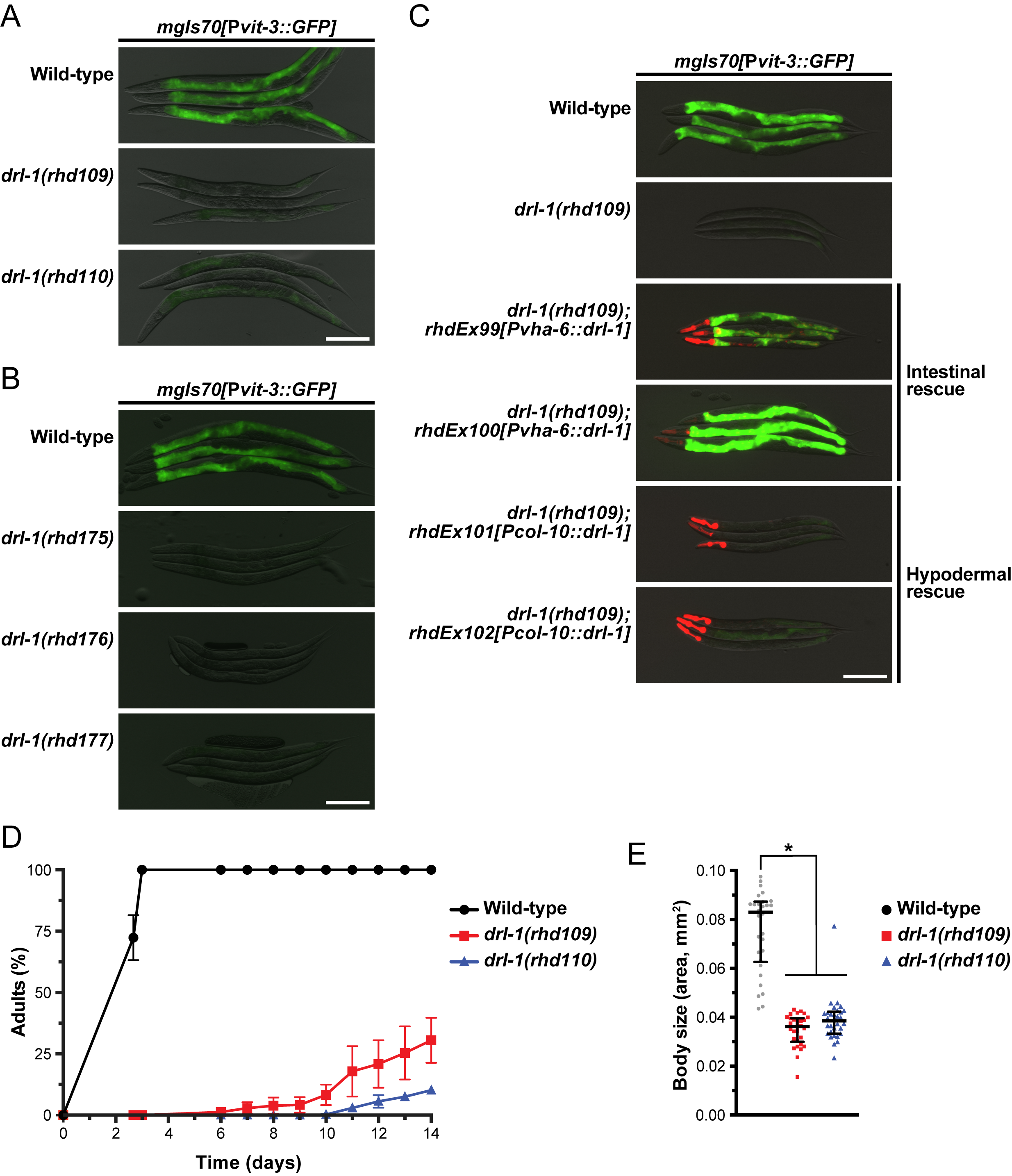

Supplement: S1 Fig — (A, B) Representative fluorescence and DIC overlaid images of mgIs70[Pvit-3::GFP] reporter expression in various drl-1 mutants (scale bar, 200 μm). The mgIs70 transgene is a high-copy transgene. (C) Intestinal, but not hypodermal, rescue of drl-1(rhd109) mutants with drl-1 cDNA restores Pvit-3::GFP expression (scale bar, 200 μm). (D) Growth rate (mean +/− SEM) and (E) body size (day 1 adults; median and interquartile range; *, P < 0.0001, one-way ANOVA) of wild-type and drl-1 mutant animals. Raw data underlying panels D and E can be found in S9 Data. (TIF) [file pbio.3002320.s001.tif]

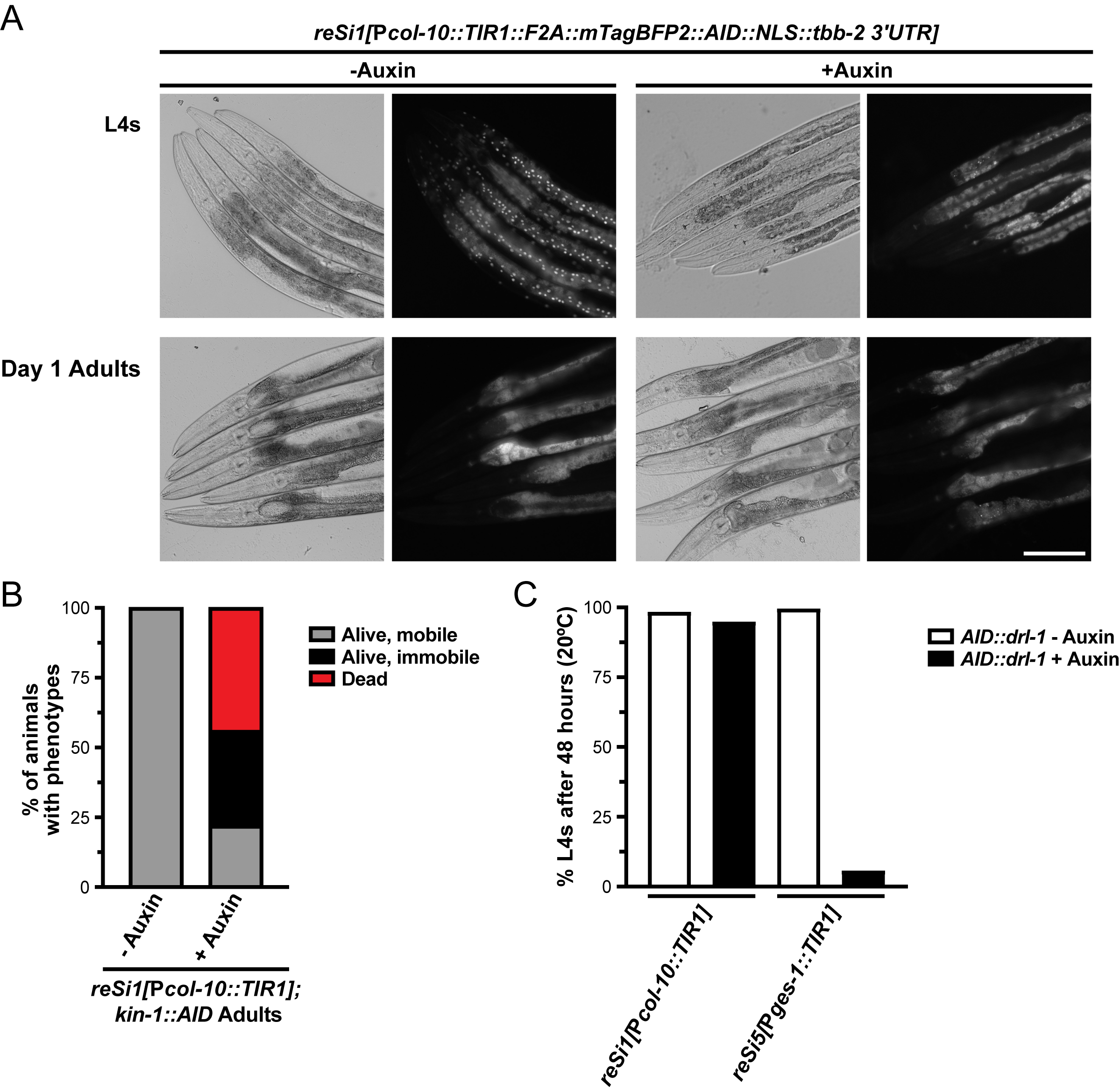

Supplement: S2 Fig — (A) Representative BFP fluorescence images of L4s and day 1 adult animals carrying the reSi1[Pcol-10::TIR1::F2A::mTagBFP2::AID*::NLS::tbb-2 3’UTR] transgene grown in the absence or presence of 4 mM auxin (scale bar, 100 μm). (B) Day 1 adult reSi1[Pcol-10::TIR1]; kin-1::AID animals treated with or without 4 mM auxin and scored 24 hours later for motility defects and/or death (n = 50 animals per condition). (C) Development of AID::drl-1 animals to the L4 stage (48 hours at 20°C) after hypodermal (reSi1) or intestinal (reSi5) depletion of DRL-1 with 4 mM auxin (n = approximately 100 per condition). Raw data underlying panels B and C can be found in S10 Data. (TIF) [file pbio.3002320.s002.tif]

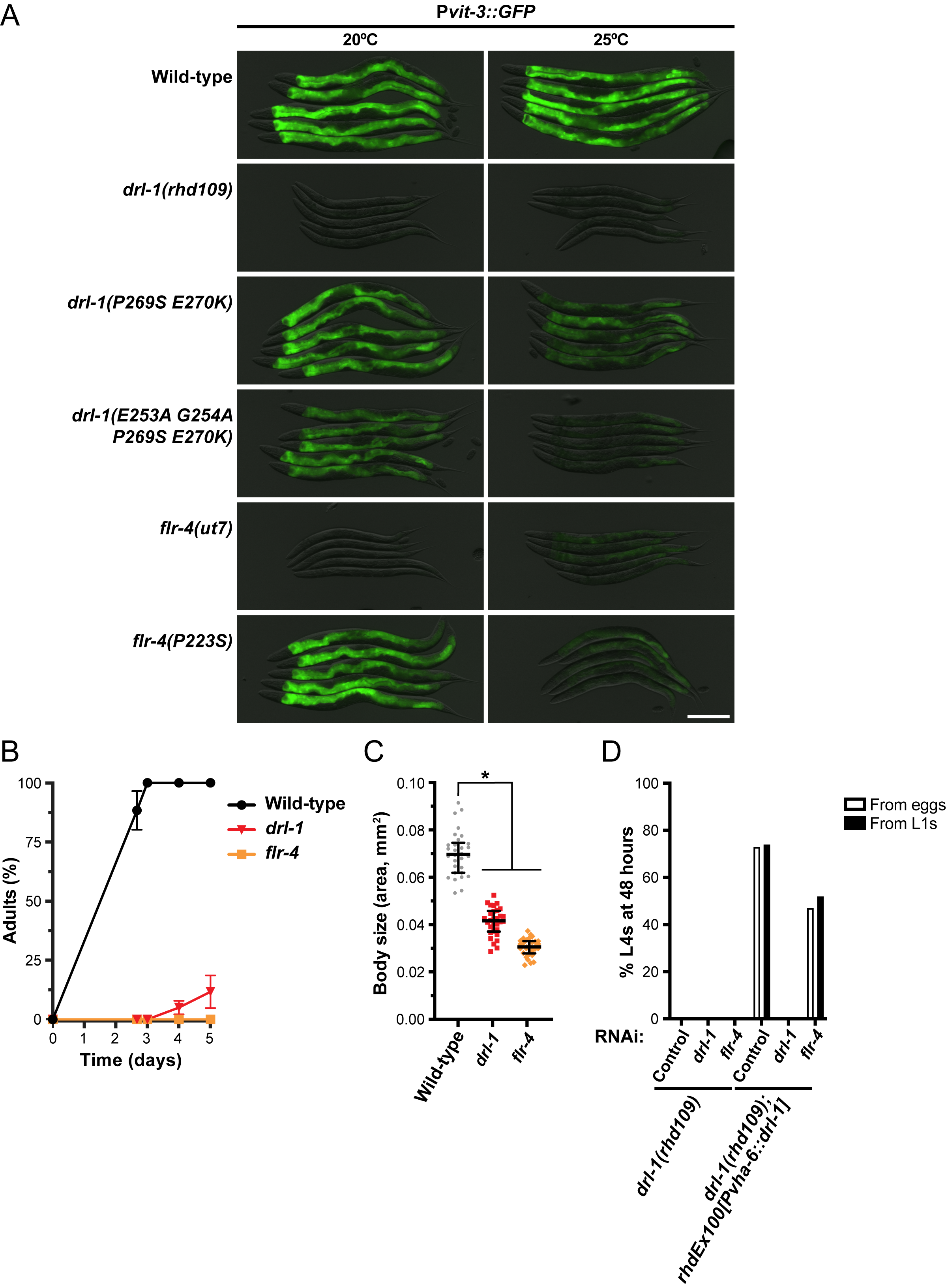

Supplement: S3 Fig — (A) Representative overlaid DIC and GFP fluorescence images of day 1 adult wild-type and mutant animals reared at 20 or 25°C (scale bar, 200 μm). (B) Growth rate (mean +/− SEM) and (C) body size (day 1 adults; median and interquartile range; *, P < 0.0001, one-way ANOVA) of wild-type, drl-1(rhd109), and flr-4(ut7) animals. (D) The percentage of animals at the L4 stage 48 hours after dropping eggs (white bars) or synchronized L1s (black bars) grown at 20°C. Animals lacking drl-1 (rhd109 allele) or overexpressing drl-1 (rhdEx100 transgenics) were subjected to control, drl-1, or flr-4 RNAi before scoring. Raw data underlying panels B, C, and D can be found in S11 Data. (TIF) [file pbio.3002320.s003.tif]

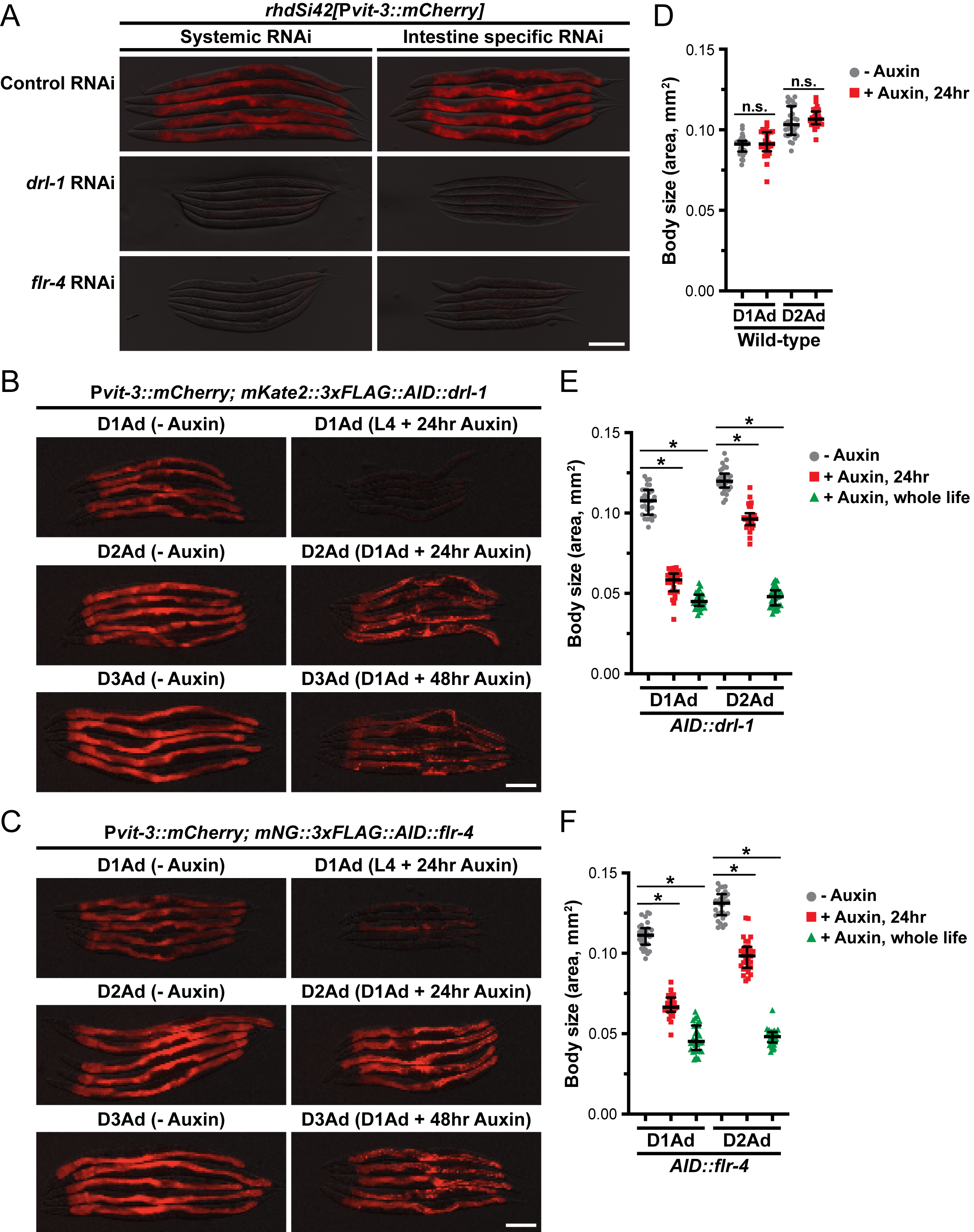

Supplement: S4 Fig — (A) Representative fluorescence images of Pvit-3::mCherry reporter expression in day 1 adult animals after whole-body or tissue-specific knockdown of drl-1 or flr-4 by RNAi (scale bar, 200 μm). Images of Pvit-3::mCherry expression after adult-specific, intestinal depletion of (B) mKate2::3xFLAG::AID::drl-1 or (C) mNG::3xFLAG::AID::flr-4 with 4 mM auxin. (B, C) Auxin was applied to L4s for 24 hours (top), day 1 adults for 24 hours (middle), or day 1 adults for 48 hours (bottom) prior to imaging alongside of the no auxin controls (scale bars, 200 μm). Body size measurements of (D) wild-type, (E) AID::drl-1, and (F) AID::flr-4 animals treated with 4 mM auxin as L4s and imaged as day 1 adults (left, D1Ad) or treated as day 1 adults and imaged as day 2 adults (right, D2Ad). For (E, F), whole life auxin treatments are also included. (D-F) All data are plotted as the median and interquartile range (n.s., not significant, *, P < 0.0001, one-way ANOVA). (B-F) All animals carry the Pges-1::TIR1 transgene. Raw data underlying panels D, E, and F can be found in S12 Data. (TIF) [file pbio.3002320.s004.tif]

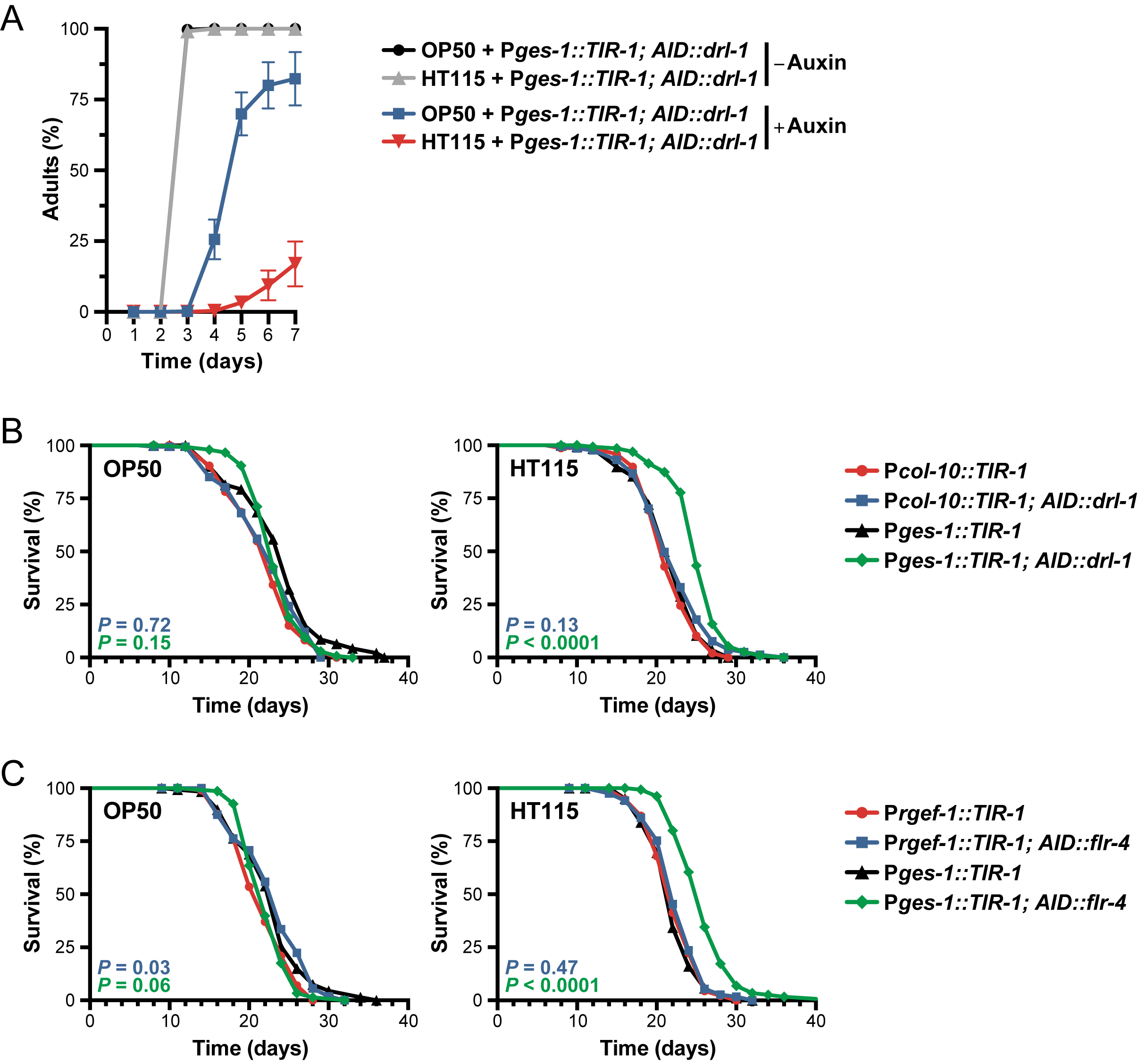

Supplement: S5 Fig — (A) Growth rate (mean +/− SEM) of mKate2::3xFLAG::AID::drl-1 animals with or without 4 mM auxin reared on E. coli OP50 or HT115. Longitudinal life span assays of (B) mKate2::3xFLAG::AID::drl-1 or (C) mNG::3xFLAG::AID::flr-4 animals grown at 20°C with FUDR on E. coli OP50 or HT115 (Pges-1::TIR1, intestinal depletion; Pcol-10::TIR1, hypodermal depletion; Prgef-1::TIR1, pan-neuronal depletion). Control animals only carry the TIR1 transgenes, and all strains were reared on 4 mM auxin from hatching. Raw data underlying panels A, B, and C can be found in S13 Data. (TIF) [file pbio.3002320.s005.tif]

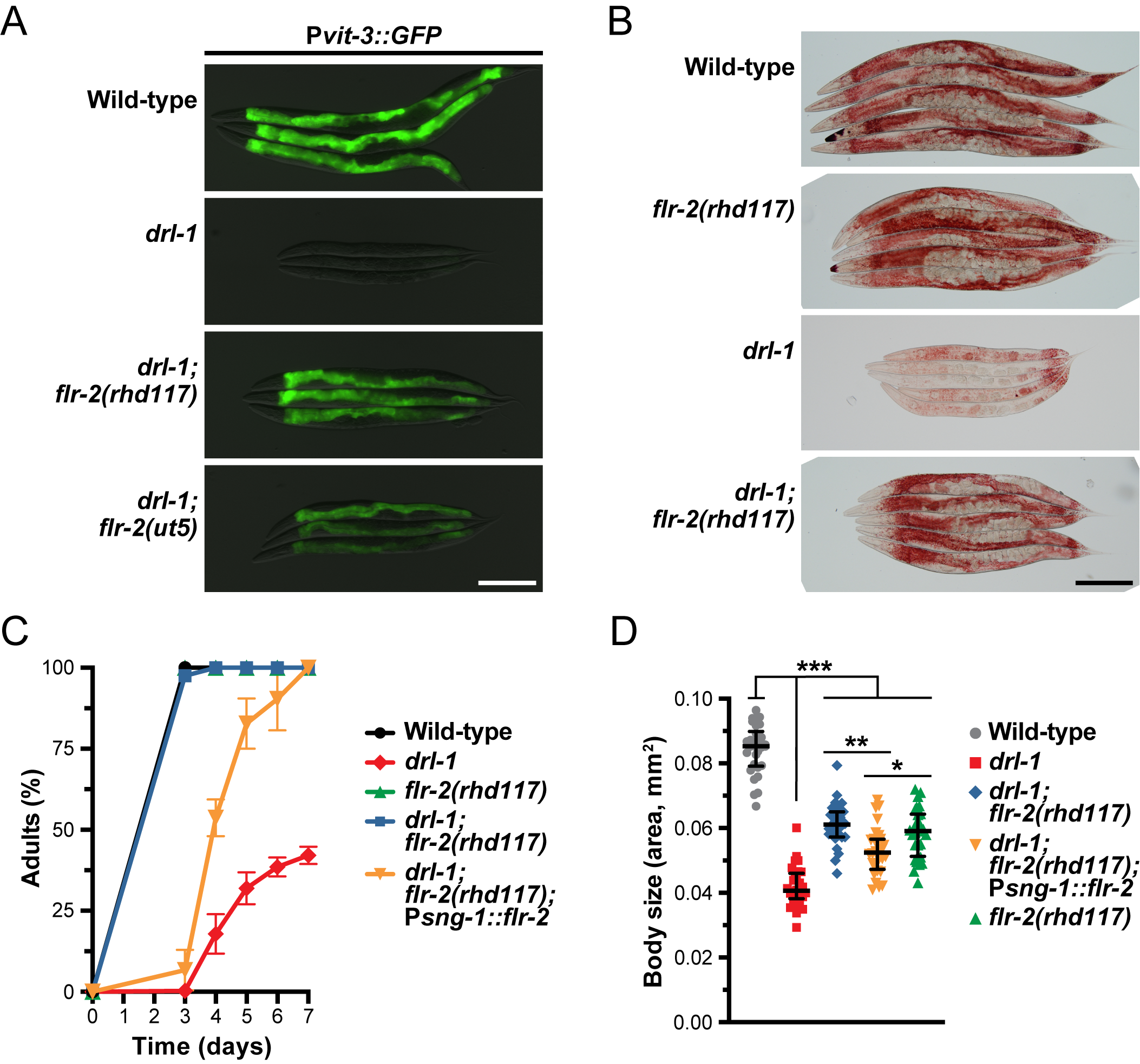

Supplement: S6 Fig — (A) Overlaid DIC and GFP fluorescence images of day 1 adult wild-type, drl-1(rhd109), and drl-1(rhd109) double mutant animals (scale bar, 200 μm). (B) Representative images of day 1 adult wild-type, flr-2(rhd117), drl-1(rhd109), and drl-1(rhd109); flr-2(rhd117) animals stained with Oil Red O (scale bar, 200 μm). (C) Growth rate (mean +/− SEM) and (D) body size (day 1 adults) of wild-type, drl-1(rhd109) single and double mutants, and flr-2 pan-neuronal rescue animals (Psng-1::flr-2 is a single-copy rescue transgene). (D) Body size data are presented as the median and interquartile range (***, P < 0.0001, **, P = 0.0003, *, P = 0.04, one-way ANOVA). Wild-type animals have a significantly larger body size compared to all other strains (P < 0.0001). Raw data underlying panels C and D can be found in S14 Data. (TIF) [file pbio.3002320.s006.tif]

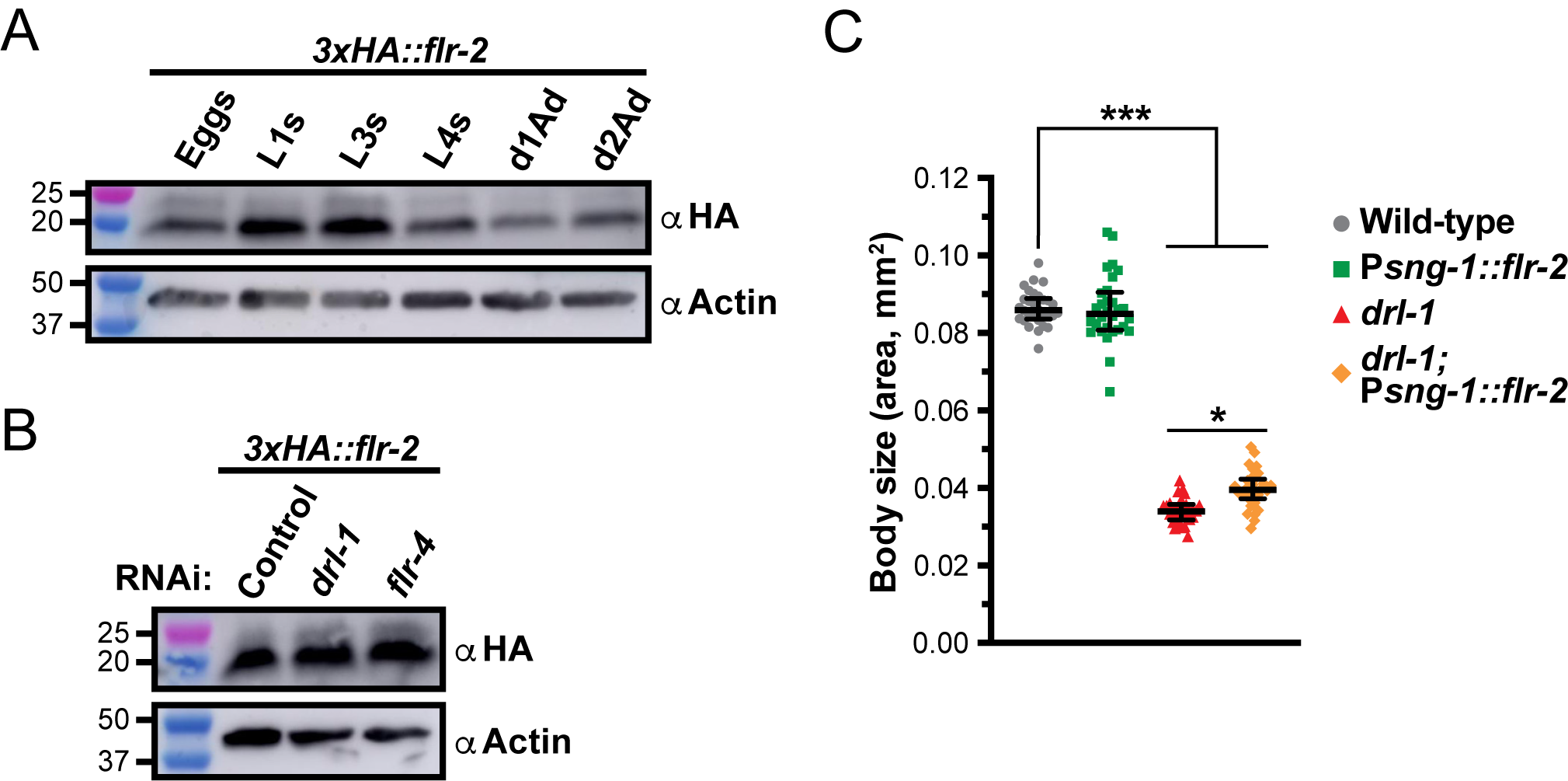

Supplement: S7 Fig — (A) A developmental time course of 3xHA::FLR-2 protein expression determined by western blotting of whole worm extracts. (B) Protein levels of 3xHA::FLR-2 after knockdown of drl-1 or flr-4 by RNAi. (B, C) The HA tag is inserted directly downstream of the signal peptide cleavage site and the faint upper bands in the HA blots are likely unprocessed FLR-2 protein. Western blot experiments were performed twice with similar results. (C) Body size data showing that overexpression of flr-2 (Psng-1::flr-2) only modestly suppresses the drl-1(rhd109) mutation (day 1 adults, ***, P < 0.0001, *, P = 0.0012, one-way ANOVA). Raw data underlying panel C can be found in S15 Data, and raw images for panels A and B can be found in S2 and S3 Raw Images, respectively. (TIF) [file pbio.3002320.s007.tif]

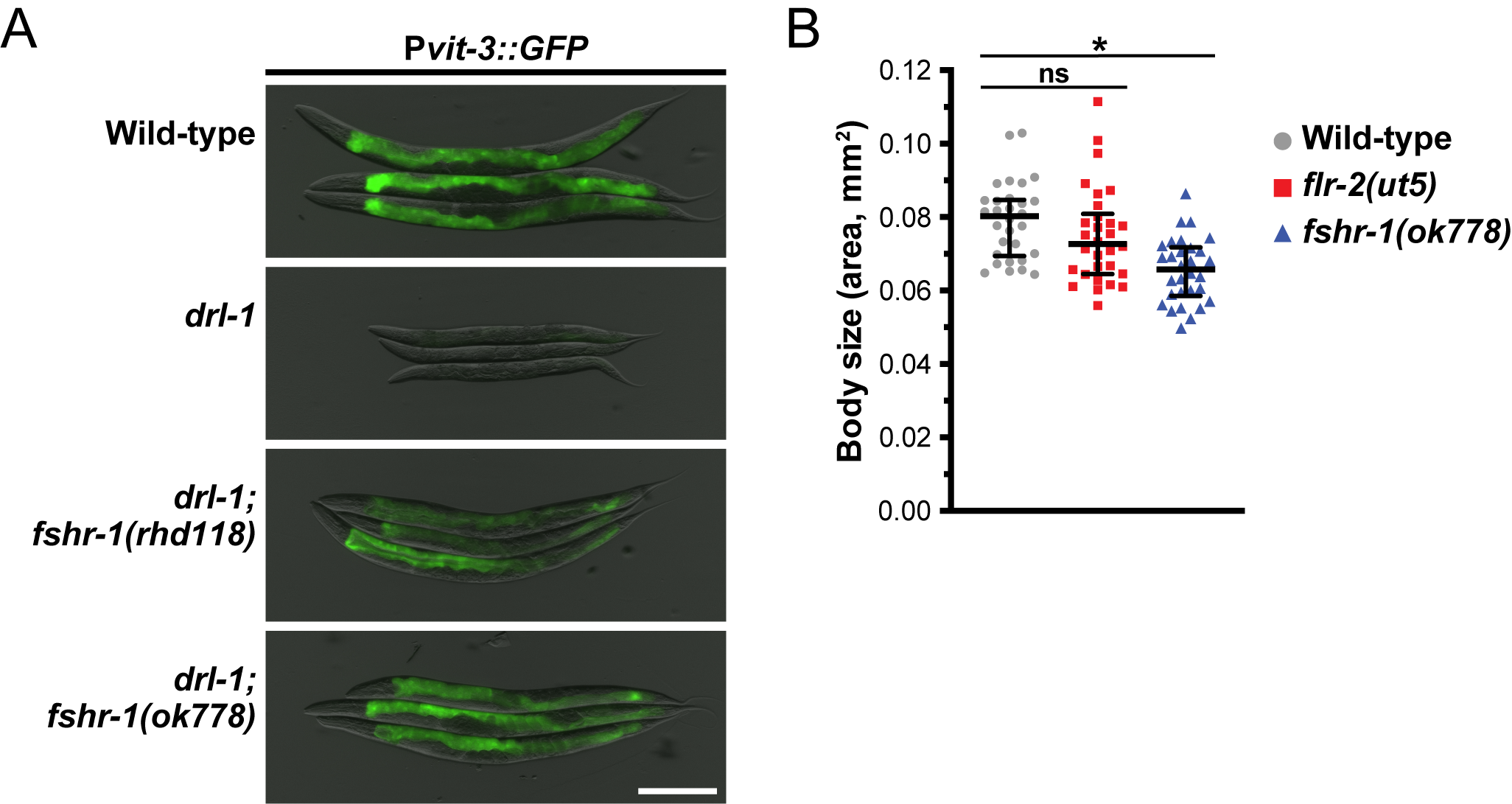

Supplement: S8 Fig — (A) Representative overlaid DIC and GFP fluorescence images of day 1 adult wild-type, drl-1(rhd109), and drl-1(rhd109); fshr-1 double mutant animals (scale bar, 200 μm). (B) Body size of day 1 adult wild-type, flr-2(ut5), and fshr-1(ok778) animals showing that both mutants are modestly smaller than wild-type (*, P < 0.0001, ns, not significant, one-way ANOVA). Raw data underlying panel B can be found in S16 Data. (TIF) [file pbio.3002320.s008.tif]

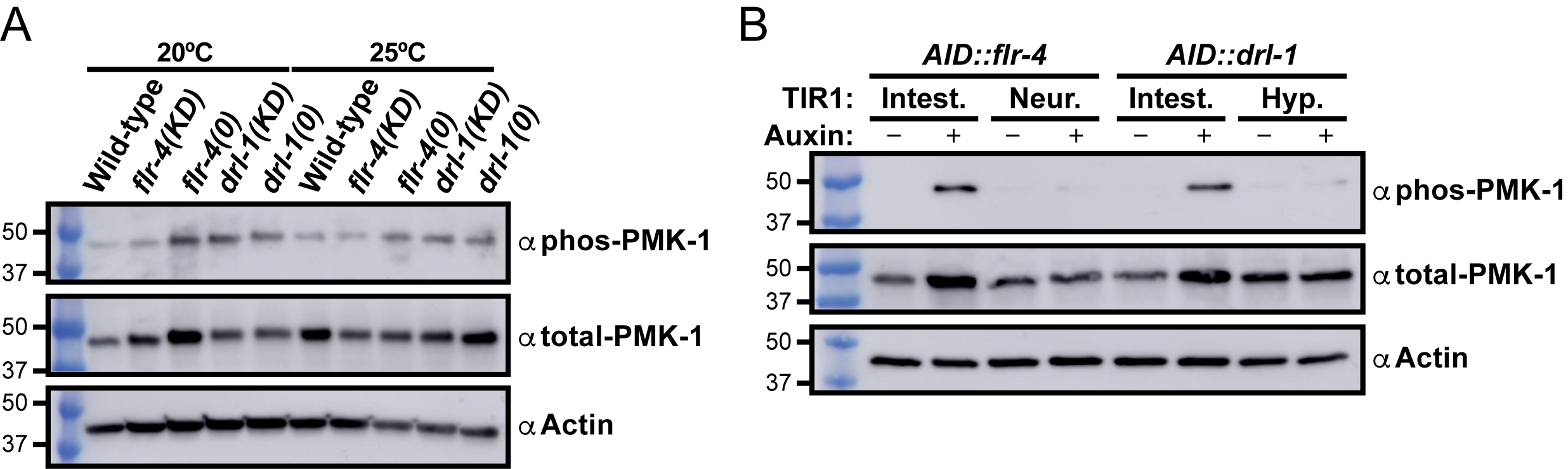

Supplement: S9 Fig — (A) Western blot analysis of phospho-PMK-1, total-PMK-1, and actin levels in wild-type, presumptive kinase dead (KD) mutants, and null (0) mutants reared at 20 or 25°C. (B) Western blot analysis of phospho-PMK-1, total-PMK-1, and actin levels in AID::drl-1 or AID::flr-4 animals grown with or without 4 mM auxin (Intest., intestinal depletion, Pges-1::TIR1; Hyp., hypodermal depletion, Pcol-10::TIR1; Neur., pan-neuronal depletion; Prgef-1::TIR1). The AID::drl-1 and AID::flr-4 strains contain the rhdSi42 transgene. Western blot experiments were performed twice with similar results. Raw images for panels A and B can be found in S4 and S5 Raw Images, respectively. (TIF) [file pbio.3002320.s009.tif]

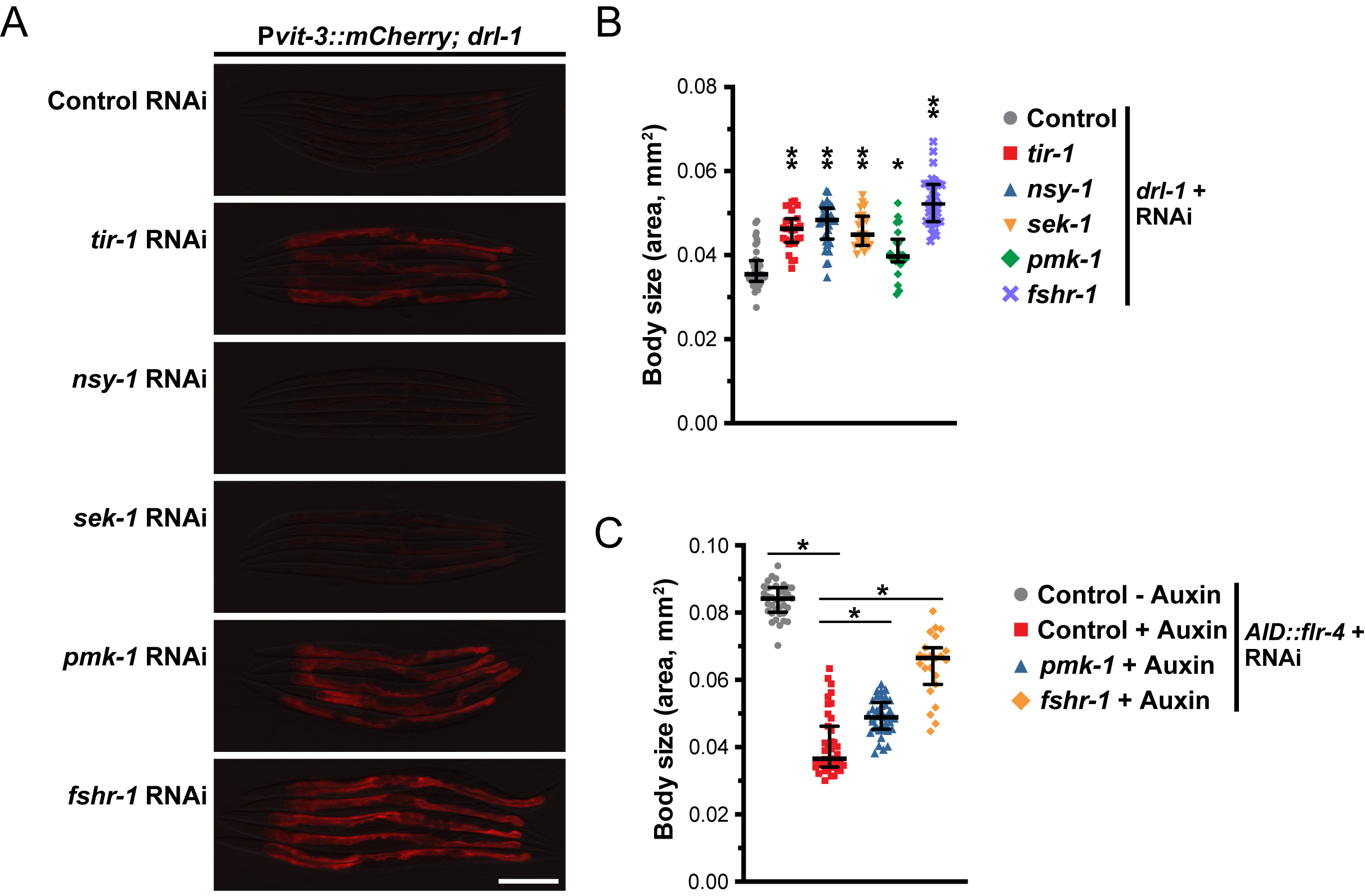

Supplement: S10 Fig — (A) Representative overlaid DIC and mCherry fluorescence images (scale bar, 200 μm) and (B) body size (median and interquartile range; *, P < 0.02, **, P < 0.001, one-way ANOVA) of day 1 adult drl-1(rhd109) animals after knockdown p38/PMK-1 pathway components by RNAi. (C) Body size of Pges-1::TIR1; mNG::3xFLAG::AID::flr-4 animals after simultaneous depletion of intestinal AID::FLR-4 (with 4 mM auxin) and knockdown of pmk-1 or fshr-1 by RNAi. Data are shown as the median and interquartile range (*, P < 0.0001, one-way ANOVA). Raw data underlying panels B and C can be found in S17 Data. (TIF) [file pbio.3002320.s010.tif]

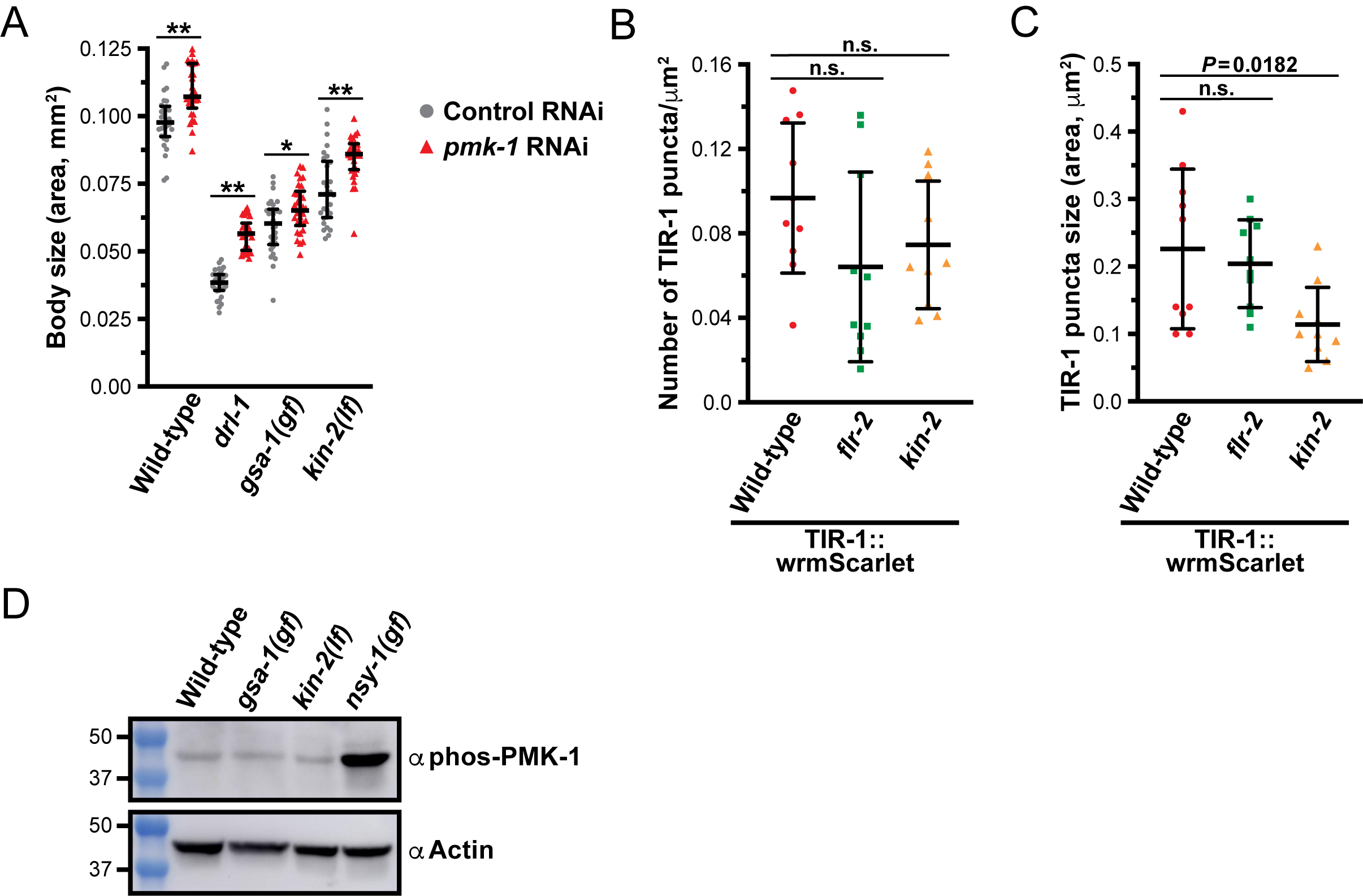

Supplement: S11 Fig — (A) Body size of wild-type animals and the indicated mutants after control or pmk-1 RNAi (day 1 adults; median and interquartile range; *, P = 0.01, **, P < 0.0001, unpaired t test). These mutations have been previously shown to activate PKA signaling (lf, loss-of-function; gf, gain-of-function). Quantification of the (B) total number and (C) absolute size of TIR-1 puncta in day 1 adult wild-type and mutant animals (mean +/− SD; n.s., not significant, one-way ANOVA). (D) A phospho-PMK-1 western blot analysis of whole animal lysates from wild-type animals and the indicated mutants. Actin was used as a loading control and the experiment was performed twice with similar results. Raw data underlying panels A, B, and C can be found in S18 Data, and raw images for panel D can be found in S6 Raw Images. (TIF) [file pbio.3002320.s011.tif]

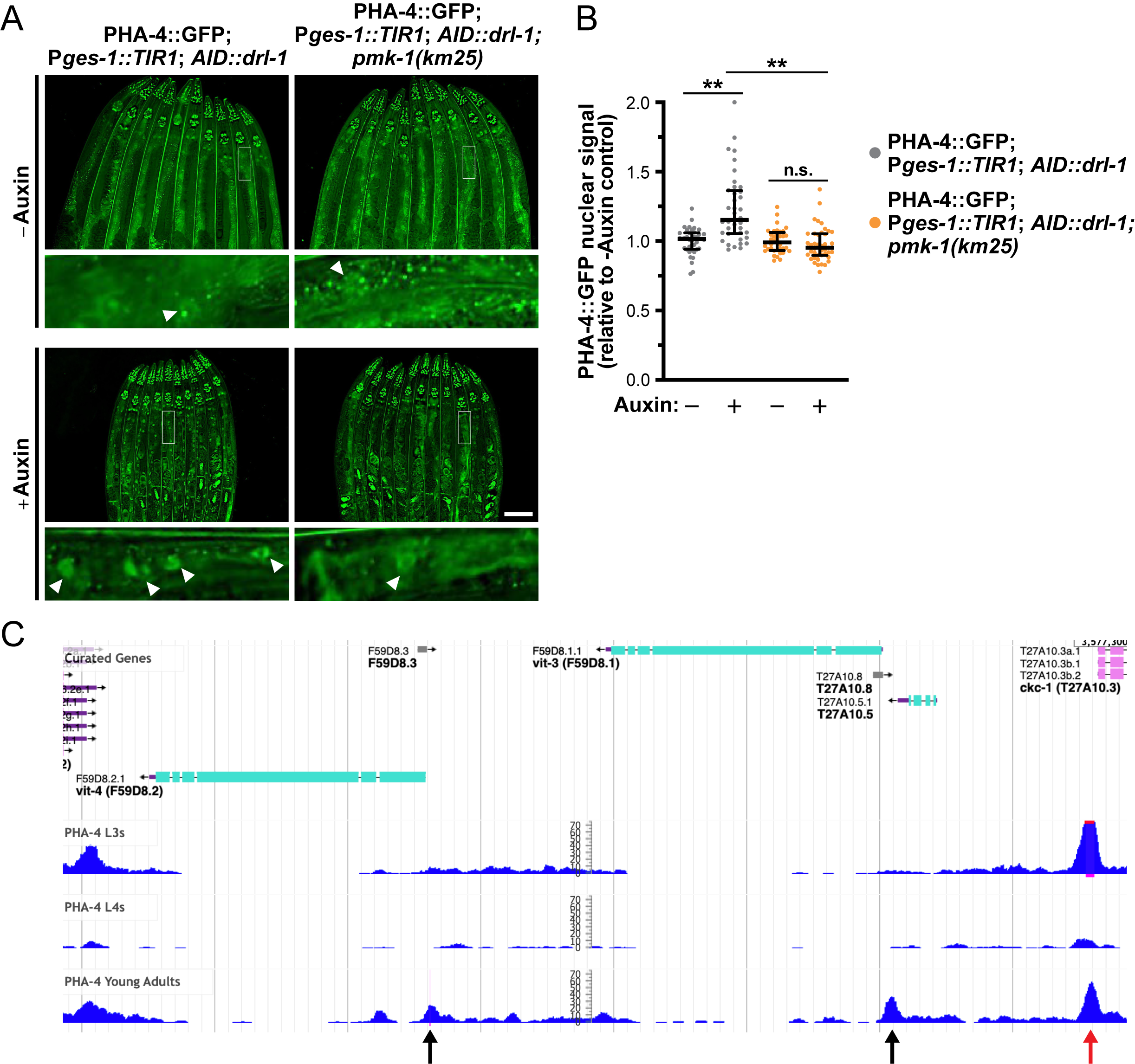

Supplement: S12 Fig — (A) Fluorescence images (white arrowheads indicate intestinal nuclei; scale bar, 100 μm) and (B) quantification (median and interquartile range; n.s., not significant, **, P < 0.0001, one-way ANOVA) of PHA-4::GFP nuclear localization after depletion of intestinal AID::DRL-1 using 4 mM auxin in wild-type or pmk-1(km25) animals grown on E. coli HT1115. (C) A screenshot of PHA-4 ChIP-Seq data generated by the modENCODE project showing moderate binding of PHA-4 to the promoters of the vit-3 and vit-4 genes (black arrows) and strong binding of PHA-4 to the downstream gene ckc-1 (red arrow). Raw data underlying panel B can be found in S19 Data. (TIF) [file pbio.3002320.s012.tif]

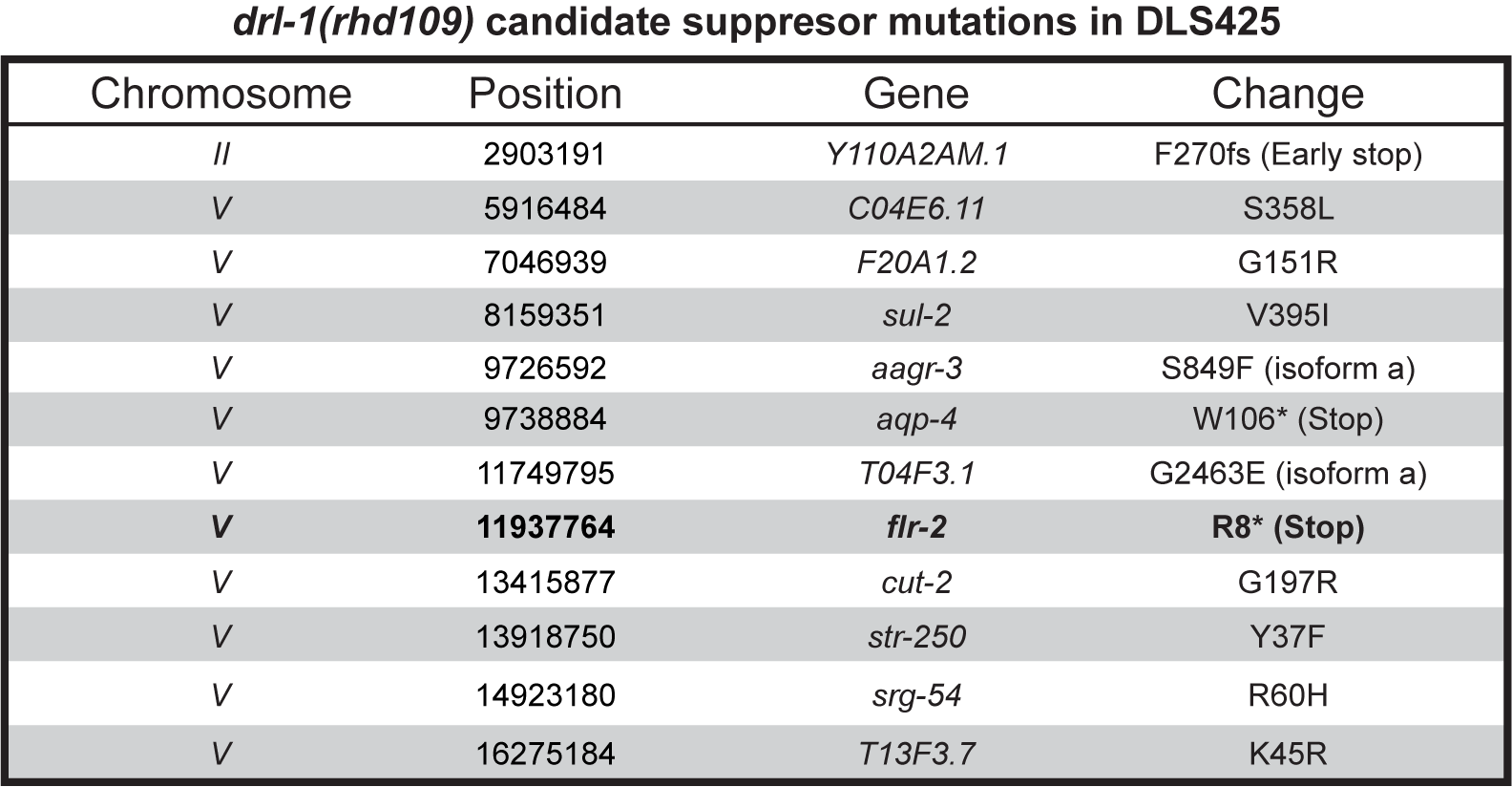

Supplement: S1 Table — To identify the causative drl-1(rhd109) suppressor mutations, EMS mutants were backcrossed to DLS364, the independently segregating F2 animals displaying the suppression phenotypes were pooled, the genomic DNA was sequenced, and candidate mutations were identified as described in the Materials and methods. The flr-2 mutation (shown in bold) was selected for further analysis since it is predicted to be a strong loss-of-function allele. The resulting amino acid change is listed in the last column. (TIF) [file pbio.3002320.s013.tif]

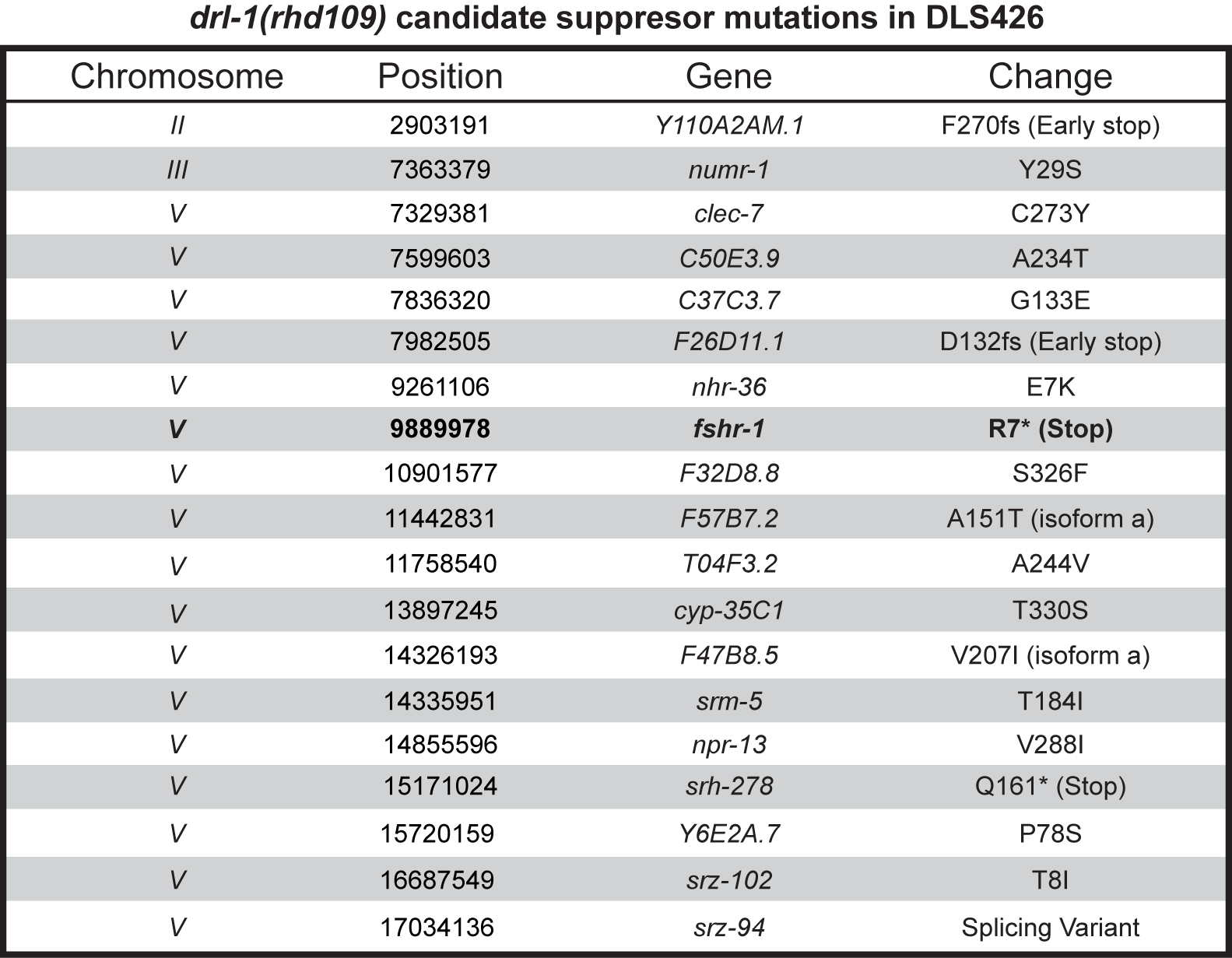

Supplement: S2 Table — The candidate causative drl-1(rhd109) suppressor mutations were identified as described in S1 Table and the Materials and methods. The fshr-1 mutation (shown in bold) was selected for further analysis since it is predicted to be a strong loss-of-function allele. The resulting amino acid change is listed in the last column. (TIF) [file pbio.3002320.s014.tif]

Fig S9A Raw Images

Day 1 adults;  
Western blot of whole  
cell lysates; images  
of chemiluminescence

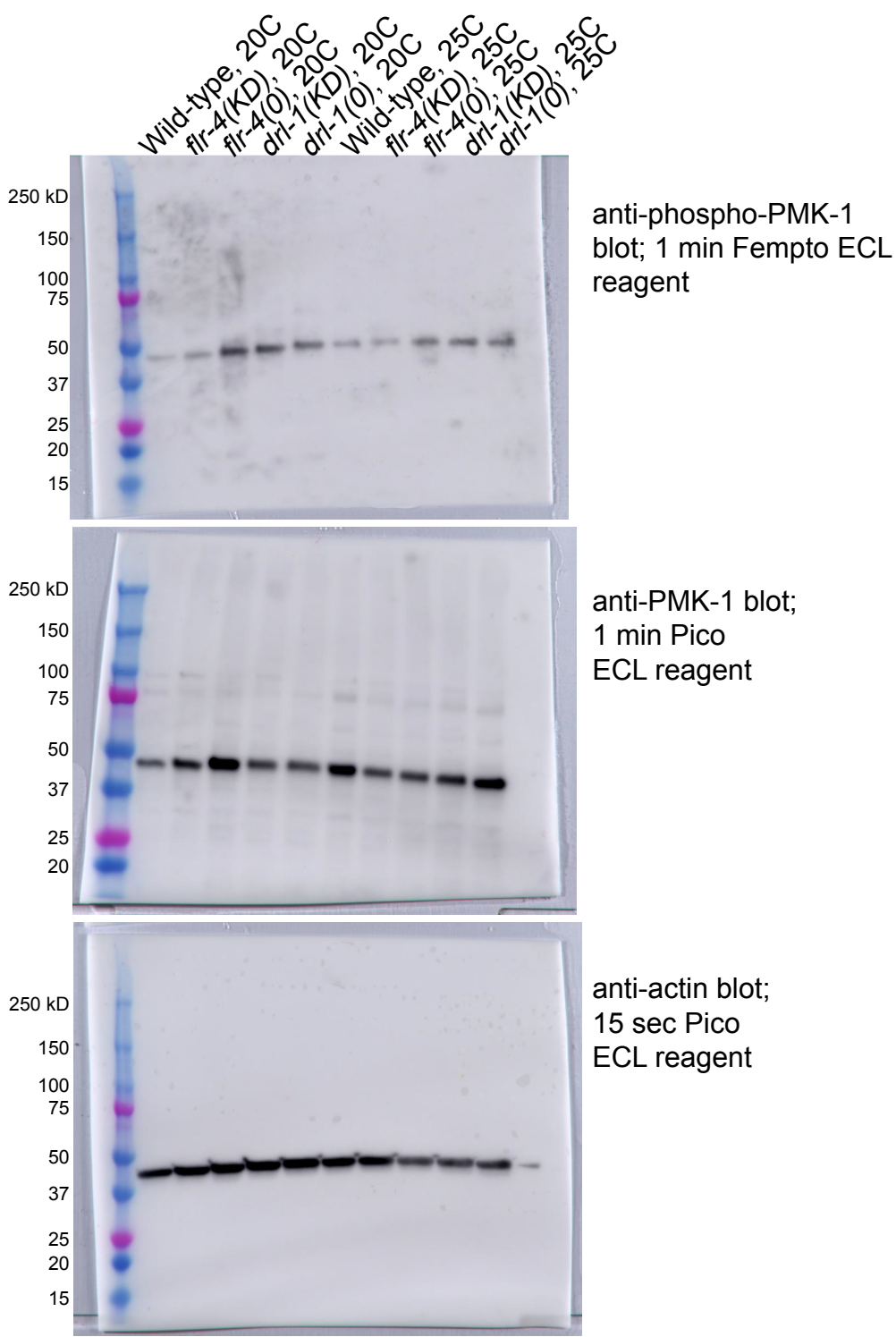

Supplement: S4 Raw Images — (PDF) [file pbio.3002320.s038.pdf]

Fig S9B Raw Images

Day 1 adults;  
Western blot of whole  
cell lysates; images  
of chemiluminescence

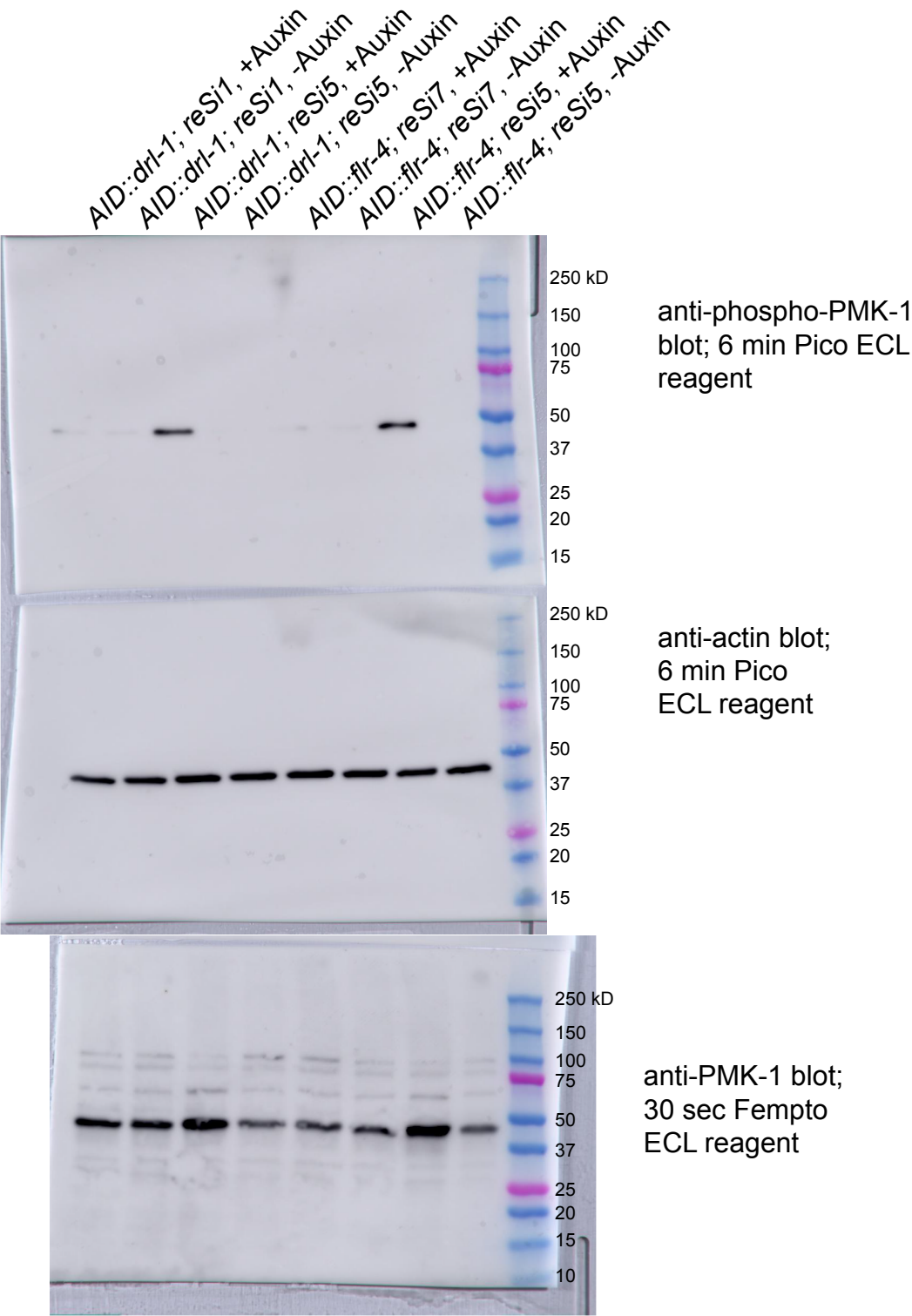

Supplement: S5 Raw Images — (PDF) [file pbio.3002320.s039.pdf]

Fig S11D Raw Images

Day 1 adults;  
Western blot of whole  
cell lysates; images  
of chemiluminescence

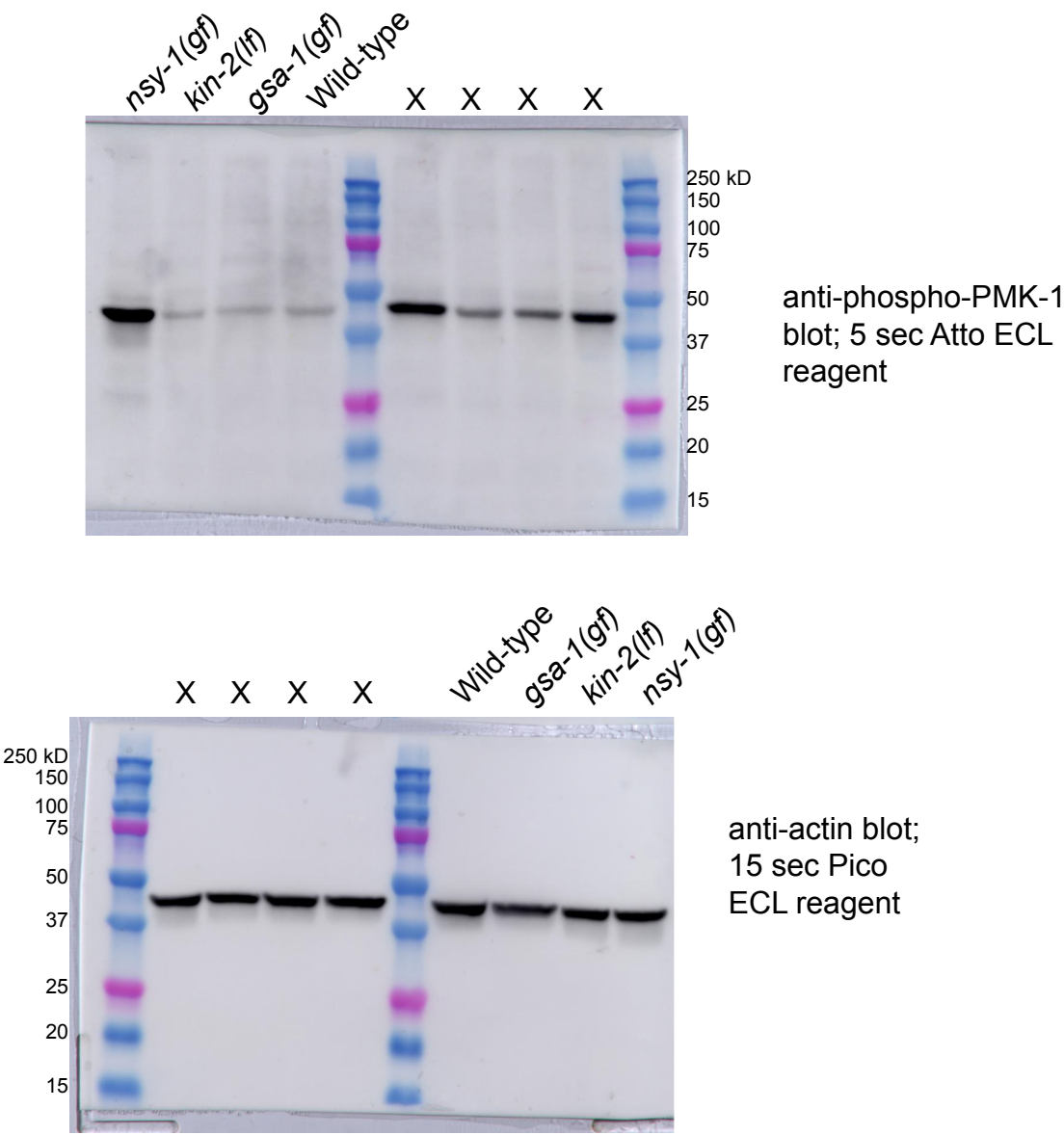

Supplement: S6 Raw Images — (PDF) [file pbio.3002320.s040.pdf]
