# Supplementary material for: Opposing action of the FLR-2 glycoprotein hormone and DRL-1/FLR-4 MAP kinases balance p38-mediated growth and lipid homeostasis in C. elegans
Source: PLoS Biol. 2023 Sep 29;21(9):e3002320. doi: 10.1371/journal.pbio.3002320 (PMC10566725; doi:10.1371/journal.pbio.3002320)
Supplement: S1 File — (PDF) [file pbio.3002320.s015.pdf]

**Strain**	**Genotype**	**Reference**
N2	Wild-type	Brenner 1974
DLS362	*drl-1(rhd109) IV*	This study
DLS363	*drl-1(rhd110) IV*	This study
DLS364	*drl-1(rhd109) IV; mgIs70[*P*vit-3::GFP]*	This study
DLS365	*drl-1(rhd110) IV; mgIs70[*P*vit-3::GFP]*	This study
DLS425	*drl-1(rhd109) IV; flr-2(rhd117) V; mgIs70[*P*vit-3::GFP]*	This study
DLS426	*drl-1(rhd109) IV; fshr-1(rhd118) V; mgIs70[*P*vit-3::GFP]*	This study
DLS428	*nhr-49(ok2165) I; drl-1(rhd109) IV*	This study
DLS513	*drl-1(rhd109) IV; mgIs70[*P*vit-3::GFP]; rhdEx99[*P*vha-6::mCherry::his-58::SL2::drl-1 cDNA]*	This study
DLS514	*drl-1(rhd109) IV; mgIs70[*P*vit-3::GFP]; rhdEx100[*P*vha-6::mCherry::his-58::SL2::drl-1 cDNA]*	This study
DLS515	*drl-1(rhd109) IV; mgIs70[*P*vit-3::GFP]; rhdEx101[*P*col-10::mCherry::his-58::SL2::drl-1 cDNA]*	This study
DLS516	*drl-1(rhd109) IV; mgIs70[*P*vit-3::GFP]; rhdEx102[*P*col-10::mCherry::his-58::SL2::drl-1 cDNA]*	This study
DLS519	*drl-1(rhd109) IV; flr-2(ut5) V; mgIs70[*P*vit-3::GFP]*	This study
DLS520	*drl-1(rhd109) IV; fshr-1(ok778) V; mgIs70[*P*vit-3::GFP]*	This study
DLS523	*flr-4(ut7) X; mgIs70[*P*vit-3::GFP]*	This study
DLS537	*rhdSi42[*P*vit-3::mCherry::unc-54 3'UTR + cb-unc-119(+)] II*	This study
DLS539	*rhdSi42[*P*vit-3::mCherry::unc-54 3'UTR + cb-unc-119(+)] alxIs9[*P*vha-6::SID-1::SL2::GFP] II; sid-1(qt9) V*	This study
DLS581	*drl-1(rhd175) IV; mgIs70[*P*vit-3::GFP]*	This study
DLS582	*drl-1(rhd176) IV; mgIs70[*P*vit-3::GFP]*	This study
DLS583	*drl-1(rhd177) IV; mgIs70[*P*vit-3::GFP]*	This study
DLS586	*drl-1(rhd180[P269S E270K]) IV; mgIs70[*P*vit-3::GFP]*	This study
DLS622	*reSi5[*P*ges-1::TIR1::F2A::mTagBFP2::NLS::AID::tbb-2 3'UTR] I; rhdSi42[*P*vit-3::mCherry::unc-54 3'UTR + cb-unc-119(+)] II*	This study
DLS626	*flr-4(n2259) X; mgIs70[*P*vit-3::GFP]*	This study
DLS627	*rhdSi42[*P*vit-3::mCherry::unc-54 3'UTR + cb-unc-119(+)] II; fshr-1(rhd118) V*	This study
DLS628	*drl-1(rhd197[E253A G254A P269S E270K]) IV; mgIs70[*P*vit-3::GFP]*	This study
DLS632	*drl-1(rhd197[E253A G254A P269S E270K]) IV*	This study
DLS634	*rhdSi42[*P*vit-3::mCherry::unc-54 3'UTR + cb-unc-119(+)] II; drl-1(rhd109) IV; fshr-1(rhd118) V*	This study
DLS635	*rhdSi42[*P*vit-3::mCherry::unc-54 3'UTR + cb-unc-119(+)] II; drl-1(rhd109) IV; flr-2(rhd117) V*	This study
DLS636	*rhdSi42[*P*vit-3::mCherry::unc-54 3'UTR + cb-unc-119(+)] II; drl-1(rhd109) IV*	This study
DLS637	*rhdSi42[*P*vit-3::mCherry::unc-54 3'UTR + cb-unc-119(+)] II; drl-1(rhd110) IV*	This study
DLS638	*rhdSi42[*P*vit-3::mCherry::unc-54 3'UTR + cb-unc-119(+)] II; flr-4(ut7) X*	This study

DLS644	*reSi5[*P*ges-1::TIR1::F2A::mTagBFP2::NLS::AID::tbb-2 3'UTR] I; rhdSi42[*P*vit-3::mCherry::unc-54 3'UTR + cb-unc-119(+)] II; drl-1(rhd203[mKate2::TEV::3xFLAG::AID::drl-1]) IV*	This study
DLS657	*rhdSi42[*P*vit-3::mCherry::unc-54 3'UTR + cb-unc-119(+)] II; flr-2(rhd117) V; flr-4(ut7) X*	This study
DLS658	*rhdSi42[*P*vit-3::mCherry::unc-54 3'UTR + cb-unc-119(+)] II; fshr-1(rhd118) V; flr-4(ut7) X*	This study
DLS663	*rhdSi42[*P*vit-3::mCherry::unc-54 3'UTR + cb-unc-119(+)] II; flr-2(rhd117) V*	This study
DLS664	*flr-2(rhd117) V*	This study
DLS665	*drl-1(rhd109) IV; flr-2(rhd117) V*	This study
DLS674	*reSi5[*P*ges-1::TIR1::F2A::mTagBFP2::NLS::AID::tbb-2 3'UTR] I; rhdSi42[*P*vit-3::mCherry::unc-54 3'UTR + cb-unc-119(+)] II; flr-4(rhd209[mNG::TEV::3xFLAG::AID::flr-4]) X*	This study
DLS685	*nhr-49(nr2041) I; rhdSi42[*P*vit-3::mCherry::unc-54 3'UTR + cb-unc-119(+)] II; drl-1(rhd109) IV*	This study
DLS686	*rhdSi46[*P*sng-1::mCherry::his-58::SL2::flr-2 + cb-unc-119(+)] II; unc-119(ed3) III*	This study
DLS696	*reSi7[*P*rgef-1::TIR1::F2A::mTagBFP2::AID::NLS::tbb-2 3'UTR] I; rhdSi42[*P*vit-3::mCherry::unc-54 3'UTR + cb-unc-119(+)] II*	This study
DLS699	*reSi7[*P*rgef-1::TIR1::F2A::mTagBFP2::AID::NLS::tbb-2 3'UTR] I; rhdSi42[*P*vit-3::mCherry::unc-54 3'UTR + cb-unc-119(+)] II; flr-4(rhd209[mNG::TEV::3xFLAG::AID::flr-4]) X*	This study
DLS700	*reSi1[*P*col-10::TIR1::F2A::mTagBFP2::AID::NLS::tbb-2 3'UTR] I; rhdSi42[*P*vit-3::mCherry::unc-54 3'UTR + cb-unc-119(+)] II; drl-1(rhd203[mKate2::TEV::3xFLAG::AID::drl-1]) IV*	This study
DLS701	*reSi1[*P*col-10::TIR1::F2A::mTagBFP2::AID::NLS::tbb-2 3'UTR] I; rhdSi42[*P*vit-3::mCherry::unc-54 3'UTR + cb-unc-119(+)] II*	This study
DLS703	*rhdSi46[*P*sng-1::mCherry::his-58::SL2::flr-2 + cb-unc-119(+)] II; drl-1(rhd109) IV*	This study
DLS712	*rhdSi42[*P*vit-3::mCherry::unc-54 3'UTR + cb-unc-119(+)] alxIs9[*P*vha-6::sid-1::SL2::GFP] II; drl-1(rhd109) IV; sid-1(qt9) V*	This study
DLS781	*drl-1(rhd203[mKate2::TEV::3xFLAG::AID::drl-1]) IV; glo-4(ok623) V; flr-4(rhd244[3xHA::mGL::flr-4]) X*	This study
DLS823	*reSi5[*P*ges-1::TIR1::F2A::mTagBFP2::NLS::AID::tbb-2 3'UTR] I; rhdSi42[*P*vit-3::mCherry::unc-54 3'UTR + cb-unc-119(+)] II; fshr-1(rhd264[fshr-1::3xFLAG::AID]) flr-2(rhd273[flr-2(1-31aa)::3xHA::flr-2(32-122)]) V*	This study
DLS831	*reSi5[*P*ges-1::TIR1::F2A::mTagBFP2::NLS::AID::tbb-2 3'UTR] I; drl-1(rhd203[mKate2::TEV::3xFLAG::AID::drl-1]) IV; pha-4(st12220[pha-4::TY1::EGFP::3xFLAG]) V*	This study
DLS835	*tir-1(ums63[tir-1::wrmScarlet]) III; drl-1(rhd109) IV; flr-2(rhd117) V*	This study
DLS836	*reSi5[*P*ges-1::TIR1::F2A::mTagBFP2::AID*::NLS::tbb-2 3'UTR] I; drl-1(rhd203[mKate2::TEV::3xFLAG::AID::drl-1]) pmk-1(km25) IV*	This study

DLS837	*rhdSi46[*P*sng-1::mCherry::his-58::SL2::flr-2 + cb-unc-119(+)] II; drl-1(rhd109) IV; flr-2(rhd117) V*	This study
DLS841	*tir-1(ums63[tir-1::wrmScarlet]) III; drl-1(rhd109) IV*	This study
DLS845	*reSi5[*P*ges-1::TIR1::F2A::mTagBFP2::AID*::NLS::tbb-2 3'UTR] I; drl-1(rhd203[mKate2::TEV::3xFLAG::AID::drl-1]) pmk-1(km25) IV; pha-4(st12220) V*	This study
DLS846	*reSi5[*P*ges-1::TIR1::F2A::mTagBFP2::AID*::NLS::tbb-2 3'UTR] I; drl-1(rhd203[mKate2::TEV::3xFLAG::AID::drl-1])*	This study
DLS848	*reSi5[*P*ges-1::TIR1::F2A::mTagBFP2::AID*::NLS::tbb-2 3'UTR] I; drl-1(rhd203[mKate2::TEV::3xFLAG::AID::drl-1]) IV; flr-2(rhd117) V*	This study
DLS887	*reSi5[*P*ges-1::TIR1::F2A::mTagBFP2::NLS::AID::tbb-2 3'UTR] I; tir-1(qd4) III; drl-1(rhd203[mKate2::TEV::3xFLAG::AID::drl-1]) IV*	This study
DLS888	*tir-1(ums63[tir-1::wrmScarlet]) III; kin-2(ce179) X*	This study
DLS895	*tir-1(ums63[tir-1::wrmScarlet]) III; flr-2(rhd117) V*	This study
DLS903	*reSi1[*P*col-10::TIR1::F2A::mTagBFP2::AID*::NLS::tbb-2 3'UTR] kin-1(rhd303[kin-1b::3xFLAG::AID kin-1a::3xFLAG::AID] I; rhdSi42[*P*vit-3::mCherry::unc-54 3'UTR + cb-unc-119(+)] II*	This study
DLS904	*reSi5[*P*ges-1::TIR1::F2A::mTagBFP2::NLS::AID::tbb-2 3'UTR] kin-1(rhd303[kin-1b::3xFLAG::AID kin-1a::3xFLAG::AID] I; rhdSi42[*P*vit-3::mCherry::unc-54 3'UTR + cb-unc-119(+)] II*	This study
DLS905	*reSi7[*P*rgef-1::TIR1::F2A::mTagBFP2::NLS::AID::tbb-2 3UTR] kin-1(rhd303[kin-1b::3xFLAG::AID kin-1a::3xFLAG::AID] I; rhdSi42[*P*vit-3::mCherry::unc-54 3'UTR + cb-unc-119(+)] II*	This study
DV3799	*reSi1[*P*col-10::TIR1::F2A::mTagBFP2::AID*::NLS::tbb-2 3'UTR]*	Ashley et al. 2021
GR2122	*mgIs70[*P*vit-3::GFP]*	Dowen et al. 2016
IG544	*nipi-3(fr4) X*	Pujol et al. 2008
JC2209	*olrn-1(ut305) X*	Torayama et al. 2007
JC49	*flr-2(ut5) V*	Take-Uchi et al. 1998
JC51	*flr-4(ut7) X*	Take-uchi et al. 2005
KG421	*gsa-1(ce81)*	Schade et al. 2005
KG532	*kin-2(ce179)*	Schade et al. 2005
MGH171	*sid-1(qt9) V; alxIs9[*P*vha-6::SID-1::SL2::GFP]*	Melo and Ruvkun 2012
MT5701	*flr-4(n2259) X*	Take-uchi et al. 2005
QK52	*rde-1(ne219) V; xkIs99[*P*wrt-2::rde-1]*	Melo and Ruvkun 2012

RB911	*fshr-1(ok778) V*	Cho et al. 2007; C. elegans Deletion Mutant Consortium 2012
RPW403	*tir-1(ums63[tir-1::wrmScarlet]) III*	Peterson et al. 2022
RPW43	*nsy-1(ums8) II; agIs44[*p*F08G5.6::GFP::unc-54(3'UTR)* + P*myo-2::mCherry]*	Cheesman et al. 2016

***C. elegans* strains used in this study.** The strain names, genotypes, and any associated references are shown.

Target Gene	Location in Gene	crRNA Sequence	Alleles Generated
drl-1	5' end	UCCGUCAAAAAUGCAUUCAGGUUUUAGAGCUAUGCU	*rhd203*
drl-1	Internal	UCUAAUGACCGGAACGCUUCGUUUUAGAGCUAUGCU	*rhd180*
drl-1	Internal	CUGGCGGAUCCUUUUAUUGAGUUUUAGAGCUAUGCU	*rhd197*
flr-2	Internal	UUAUUACAGUACUGCACAGCGUUUUAGAGCUAUGCU	*rhd273*
flr-4	5' end	UAAUUUAUUGGCAUUCCCGUGUUUUAGAGCUAUGCU	*rhd209, rhd244*
fshr-1	3' end	CAAUCAGAAACUACGAGAAGGUUUUAGAGCUAUGCU	*rhd264*
kin-1a	5' end	AGUUUUAAUUCAUUAUCUUUGUUUUAGAGCUAUGCU	*rhd303*
kin-1b	5' end	AGAGCUGUUCGUCGAGUUUUGUUUUAGAGCUAUGCU	*rhd303*

The crRNAs used in this study. A list of the crRNA guides, including their genomic targets and ribonucleotide sequences, that were used in this study. The alleles generated using CRISPR/Cas9 gene editing are also shown (far right column).

mRNA Target	Primer Sequence (5' to 3')	Reference
act-1	F: GCTGGACGTGATCTTACTGATTACC<br>R: GTAGCAGAGCTTCTCCTTGATGTC	(Hoogewijs et al. 2008)
vit-1	F: GAGGTTCGCTTTGACGGATA<br>R: GGCTTCACATTCCTCGTTCT	(Ding and Grosshans 2009)
vit-2	F: GACACCGAGCTCATCCGCCCA<br>R: TTCCTTCTCTCCATTGACCT	(DePina et al. 2011)
vit-3/4/5	F: CATGTGCACCATCGAAGAACTC<br>R: CCAATGTGGTTTCAATGACAAGTTG	(Dowen et al. 2016)
vit-6	F: TTCACCCAGAAGCCAGTTC<br>R: AGGATGGGAGGCAGTAGAC	(Dowen et al. 2016)
ech-9	F: AGGAAAATGGACTTGAGCCG<br>R: CTTTCCGTTGGGTTTTATCGTC	This study
ugt-18	F: AACCGGCACTGATAATTCCCCTTATGG<br>R: TAGAGCCCCATGTTCAACTGC	(Chamoli et al. 2014)

The RT-qPCR primers. The primer sequences (5' to 3') and any associated references are shown for the qPCR primers used in this study.

References

Ashley GE, Duong T, Levenson MT, Martinez MAQ, Johnson LC, Hibshman JD, Saeger HN, Palmisano NJ, Doonan R, Martinez-Mendez R, et al. 2021. An expanded auxin-inducible degron toolkit for *Caenorhabditis elegans*. *Genetics* **217**: iyab006.

Brenner S. 1974. The genetics of *Caenorhabditis elegans*. *Genetics* **77**: 71–94.

C. elegans Deletion Mutant Consortium. 2012. large-scale screening for targeted knockouts in the Caenorhabditis elegans genome. *G3 (Bethesda)* **2**: 1415–1425.

Chamoli M, Singh A, Malik Y, Mukhopadhyay A. 2014. A novel kinase regulates dietary restriction-mediated longevity in *Caenorhabditis elegans* . *Aging Cell* **13**: 641–655.

Cheesman HK, Feinbaum RL, Thekkiniath J, Dowen RH, Conery AL, Pukkila-Worley R. 2016. Aberrant Activation of p38 MAP Kinase-Dependent Innate Immune Responses Is Toxic to *Caenorhabditis elegans*. *G3 Genes|Genomes|Genetics* **6**: 541–549.

Cho S, Rogers KW, Fay DS. 2007. The C. elegans Glycopeptide Hormone Receptor Ortholog, FSHR-1, Regulates Germline Differentiation and Survival. *Current Biology* **17**: 203–212.

DePina AS, Iser WB, Park S-S, Maudsley S, Wilson MA, Wolkow CA. 2011. Regulation of Caenorhabditis elegans vitellogenesis by DAF-2/IIS through separable transcriptional and posttranscriptional mechanisms. *BMC Physiol* **11**: 11.

Ding XC, Grosshans H. 2009. Repression of C. elegans microRNA targets at the initiation level of translation requires GW182 proteins. *EMBO J* **28**: 213–222.

Dowen RH, Breen PC, Tullius T, Conery AL, Ruvkun G. 2016. A microRNA program in the *C. elegans* hypodermis couples to intestinal mTORC2/PQM-1 signaling to modulate fat transport. *Genes Dev* **30**: 1515–1528.

Gerstein MB, Lu ZJ, Van Nostrand EL, et al. 2010. Integrative analysis of the *Caenorhabditis elegans* genome by the modENCODE project. *Science* **330**:1775-1787.

Hoogewijs D, Houthoofd K, Matthijssens F, Vandesompele J, Vanfleteren JR. 2008. Selection and validation of a set of reliable reference genes for quantitative sod gene expression analysis in C. elegans. *BMC Mol Biol* **9**: 9.

Melo JA, Ruvkun G. 2012. Inactivation of conserved C. elegans genes engages pathogen- and xenobiotic-associated defenses. *Cell* **149**: 452–466.

Peterson ND, Icso JD, Salisbury JE, Rodríguez T, Thompson PR, Pukkila-Worley R. 2022. Pathogen infection and cholesterol deficiency activate the C. elegans p38 immune pathway through a TIR-1/SARM1 phase transition. *Elife* **11**: e74206.

Pujol N, Cypowyj S, Ziegler K, Millet A, Astrain A, Goncharov A, Jin Y, Chisholm AD, Ewbank JJ. 2008. Distinct innate immune responses to infection and wounding in the C. elegans epidermis. *Curr Biol* **18**: 481–489.

Schade MA, Reynolds NK, Dollins CM, Miller KG. 2005. Mutations That Rescue the Paralysis of Caenorhabditis elegans ric-8 (Synembryn) Mutants Activate the Gαs Pathway and Define a Third Major Branch of the Synaptic Signaling Network. *Genetics* **169**: 631–649.

Take-Uchi M, Kawakami M, Ishihara T, Amano T, Kondo K, Katsura I. 1998. An ion channel of the degenerin/epithelial sodium channel superfamily controls the defecation rhythm in Caenorhabditis elegans. *Proc Natl Acad Sci U S A* **95**: 11775–11780.

Take-uchi M, Kobayashi Y, Kimura KD, Ishihara T, Katsura I. 2005. FLR-4, a Novel Serine/Threonine Protein Kinase, Regulates Defecation Rhythm in *Caenorhabditis elegans*. *MBoC* **16**: 1355–1365.

Torayama I, Ishihara T, Katsura I. 2007. Caenorhabditis elegans integrates the signals of butanone and food to enhance chemotaxis to butanone. *J Neurosci* **27**: 741–750.
