# Supplementary material for: Opposing action of the FLR-2 glycoprotein hormone and DRL-1/FLR-4 MAP kinases balance p38-mediated growth and lipid homeostasis in C. elegans
Source: PLoS Biol. 2023 Sep 29;21(9):e3002320. doi: 10.1371/journal.pbio.3002320 (PMC10566725; doi:10.1371/journal.pbio.3002320)
Supplement: S1 Raw Images — (PDF) [file pbio.3002320.s035.pdf]

Fig 3E Raw Images

Day 1 adults expressing HA::FLR-4 and FLAG::DRL-1; A reciprocal co-IP was performed from a whole cell lysate (WCL); images of chemiluminescence

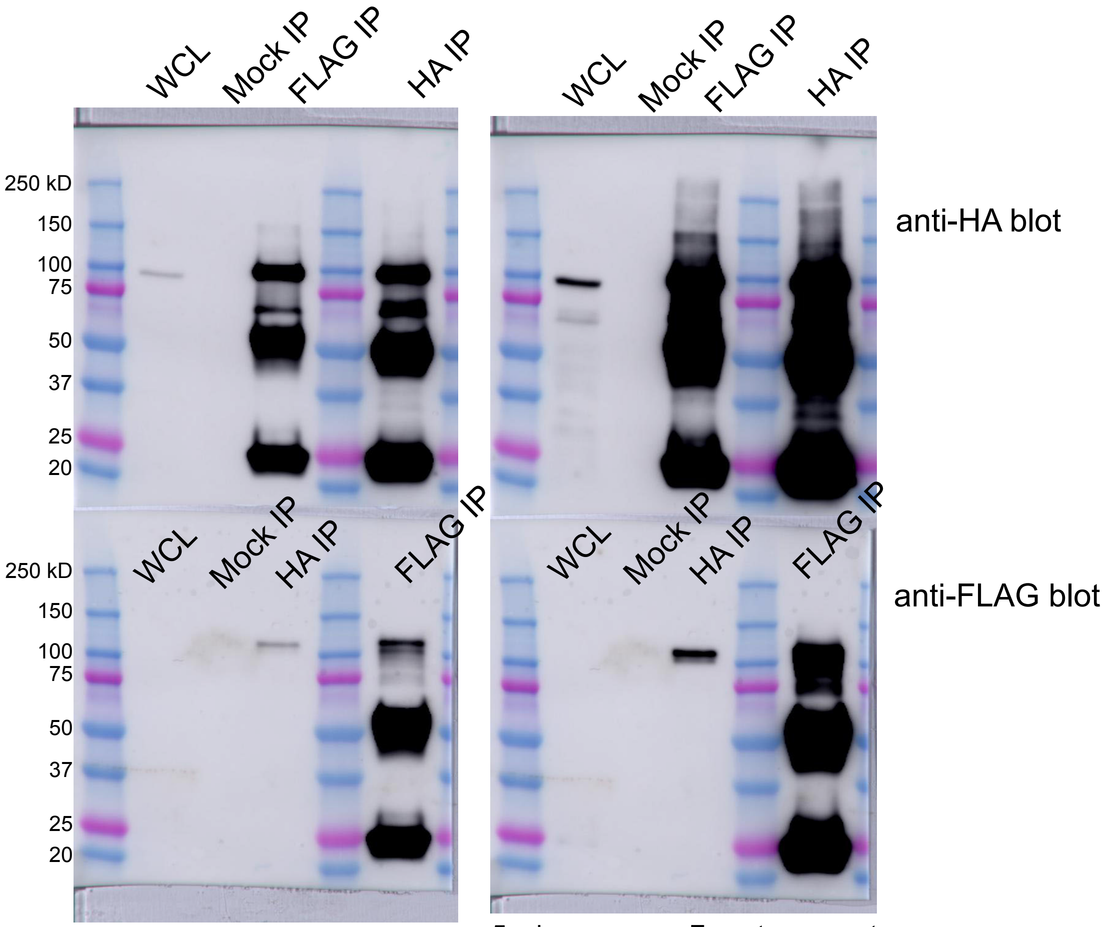

1 min exposure, Fempto reagent; Used anti-HA blot in Fig 3E

5 min exposure, Fempto reagent; Used anti-FLAG blot in Fig 3E
