# Supplementary material for: Opposing action of the FLR-2 glycoprotein hormone and DRL-1/FLR-4 MAP kinases balance p38-mediated growth and lipid homeostasis in C. elegans
Source: PLoS Biol. 2023 Sep 29;21(9):e3002320. doi: 10.1371/journal.pbio.3002320 (PMC10566725; doi:10.1371/journal.pbio.3002320)
Supplement: S2 Raw Images — (PDF) [file pbio.3002320.s036.pdf]

Fig S7A Raw Images

Animals at different developmental stages expressing HA::FLR-2 and FSHR-1::FLAG; Western blot of whole cell lysates; images of chemiluminescence

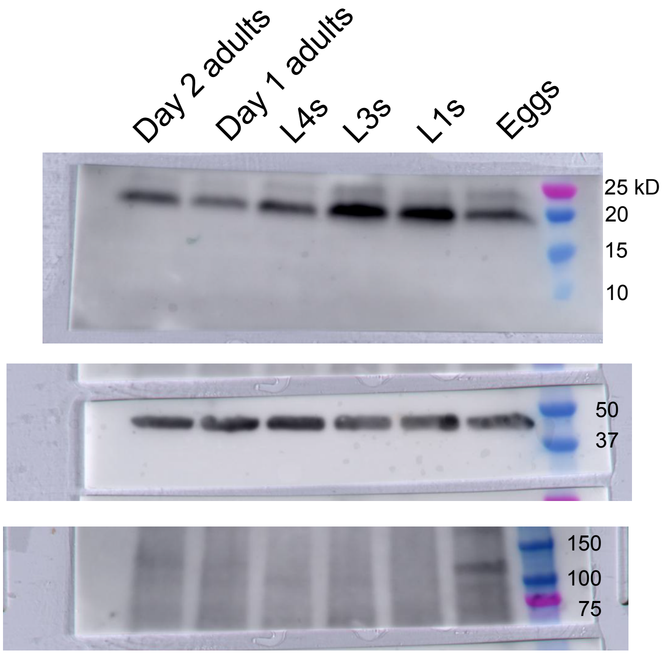

anti-HA blot; 15 sec Atto ECL reagent

anti-actin blot; 2 min, 30 sec Pico ECL reagent

anti-FLAG blot; 5 min Pico ECL reagent; FSHR-1 not clearly detected
