# Supplementary material for: Opposing action of the FLR-2 glycoprotein hormone and DRL-1/FLR-4 MAP kinases balance p38-mediated growth and lipid homeostasis in C. elegans
Source: PLoS Biol. 2023 Sep 29;21(9):e3002320. doi: 10.1371/journal.pbio.3002320 (PMC10566725; doi:10.1371/journal.pbio.3002320)
Supplement: Attachment — Submitted filename: Response_to_Reviews.docx [file pbio.3002320.s041.docx]

**Response to reviewers’ comments:**

Reviewer #1:

*The manuscript by Torzone, Park, and colleagues identifies the molecular and spatial components of signaling pathways that regulate vitellogenesis in C. elegans. Vitellogenins are lipoproteins through which hermaphrodite C. elegans transport lipids to their oocytes and embryos. This manuscript builds on previous findings that DRL-1, a kinase orthologous to the mammalian MEKK3, is a regulator of vitollegenesis. The new components identified and analyzed in this manuscript are i) FLR-4, a closely related MAPK that the authors show likely physically interacts with DRL-1, ii) FLR-2, a neuropeptide, iii) FSHR-1, a GPCR with similarity to glycoprotein hormone receptors such as the Follicle Stimulating hormone, and its predicted downstream signaling components (the canonical cAMP/PKA pathway functioning downstream of a Gαs coupled receptor), and iv) PHA-4, a FOXA family transcription factor. The model proposed by the authors is that DRL-1 and FLR-4 kinases function in the C. elegans intestine, the tissue in which vitollegenins are formed, to promote expression of vit-3, encoding a subset of vitellogenin proteins and used as a marker of vitellogenesis here, along with growth rate and body size. In turn, neurally secreted FLR-2 acts on FSHR-1 to activate the intestinal PKA to counter this. They propose DRL-1/FLR-4 complex as well as PKA exert their effects by modulating TIR-1/SARM1, an upstream regulatory component of the p38 MAPK pathway. Finally, the p38 pathway is proposed to exert its effects, in part, through the PHA-4 transcription factor.

The area of investigation is interesting as there is relatively little known about the mechanisms that coordinate vitellogenesis, growth, and development. The model described above is mostly supported by genetic epistasis data as well as tissue specific rescue/knock-down experiments with several independent readouts. Additionally, there is some biochemical data to support the idea that FLR-4 and DRL-1 physically interact. Although many of the regulatory interactions remain poorly understood at a biochemical or molecular level, the conclusions are well supported by the presented data and the findings constitute a real advance for identifying new components that coordinate vitellogenesis.

Although some of the regulatory interactions of this manuscript were either already known or easily predictable (such as the role of FLR-4 and the p38 pathway), other components are novel and emerged from unbiased screens (such as the role of the FL-2/FSHR-1 pathway). Even in the context of the predicted relationships, the authors have made advances by developing reagents that allowed for more rigorous analyses (such as tagging of endogenous loci; tissue specific depletions of signaling components). Compared to the rest of the manuscript, the data pertaining to the role of PHA-4 are less convincing, but the authors appropriately describe the role of PHA-4 as being partial (inclusion of the PHA-4 data in the manuscript is justified).

Overall, the manuscript is quite solid, well-written, and, as indicated, an advance. There are a few, minor concerns with the manuscript, most of which can be remedied by changes/additions to the writing:

1) As the authors nicely describe, vitellogenesis is upregulated at a very specific stage of development. What is not very clear from the data is the temporal aspects, if any, of the described signaling pathways. The manuscript would be strengthened if such data could be provided, for example, is secretion of FLR-2 developmentally regulated? This data is not necessary for publication but would strengthen the manuscript. In the absence of data, a clearer discussion of this point should be included.*

To investigate the expression of FLR-2 during development we knocked a 3xHA epitope tag into the *flr-2* locus directly following the secretion signal cleavage site using CRISPR/Cas9 editing, which allowed us to determine the levels of FLR-2 across development. The 3xHA::FLR-2 protein (which we determined is fully functional as this allele does not suppress *drl-1* knockdown) is expressed throughout larval development, peaking near the L3 stage, and decreases into adulthood. Surprisingly, detectable levels are present at all the time points that we selected, and importantly, loss of *drl-1* or *flr-4* had no impact on FLR-2 protein levels in day 1 adults. We also attempted to profile FSHR-1::3xFLAG levels in a similar manner; however, we were unable detect FSHR-1 by Western blotting.

We have added the following text to the results section (line 321): “To explore whether *flr-2* is developmentally regulated, we inserted a HA tag into the *flr-2* locus using CRISPR/Cas9 and performed a Western blot analysis of HA::FLR-2 from lysates of animals at different developmental stages. While FLR-2 is expressed throughout larval development and into adulthood, expression peaks at the L3 stage and is lowest during reproduction (S7A Fig), suggesting that *flr-2* expression may be repressed at adulthood to facilitate vitellogenin production. Notably, knockdown of *drl-1* or *flr-4* did not alter FLR-2 levels, and constitutive over-expression of *flr-2* in neurons does not suppress the *drl-1* body size defects, which together, indicate that *flr-2* acts in parallel, and not downstream, of *drl-1* (S7B-C Figs).”.

*2) Similar to point 1, data OR a clearer discussion on the temporal roles of DRL-1/FLR-4 would be helpful: given that their losses slow down development, is the loss of vit-3 expression a secondary consequence of this slowed down development? Same applies to PHA-4.*

Given that *AID::drl-1* and *AID::flr-4* animals reared on auxin (as well as the genetic mutants) develop slowly throughout larval development, it is likely that both proteins are expressed during all larval stages. To address the question of whether loss of *vit-3* expression is simply a function of slowed development, we exposed the *AID::drl-1* and *AID::flr-4* animals to auxin as adults and inspected P*vit-3::mCherry* expression 24 or 48 hours later. Surprisingly, adult-specific depletion of AID::DRL-1 or AID::FLR-4 decreased P*vit-3::mCherry* expression and reduced body size after 24 hours (S4B-F Figs), indicating that impaired vitellogenesis is not a secondary consequence of slowed development. Given this observation, it is unlikely that *pha-4* (or any other *drl-1* suppressor) solely acts during larval development.

We have added the following text to the results section (line 233): “Surprisingly, post-developmental depletion of either DRL-1 or FLR-4 reduced P*vit-3::mCherry* reporter expression and decreased the body size of day 2 adults (S4B-F Figs), indicating that these phenotypes are not soley a consequence of impaired development and are likely driven by metabolic dysfunction.”

And this text to the discussion (line 575): “It is likely that impaired vitellogenin production is a consequence of this dietary restriction-like metabolic state and not due to impaired developmental programs, as other models of dietary restriction also display reduced vitellogenin levels [10]. Consistently, post-developmental depletion of DRL-1 or FLR-4 reduced *vit-3* reporter expression and decreased body size.”

*3) While the introduction section of the manuscript nicely described vitellogenesis and the vit-3 reporter is a key reagent in the manuscript, most of the rest of the manuscript, especially the discussion section, seems to move away from vitellogenesis and instead use the more generic "lipid homeostasis" as a way of describing the data. Of course, vitellogenesis affects "lipid homeostasis" but the authors appear to almost treat lipid homeostasis and vitellogenesis as two unrelated processes when discussing their results. The intestinal lipids are the obvious source of lipids for the vitellogenin lipoproteins. The manuscript would be improved by being clearer about this.*

We thank the Reviewer for their insightful suggestions and agree that we often use “lipid homeostasis” when describing the phenotypes in the paper. Since *drl-1* mutants have dramatic lipid storage defects, as well as impaired vitellogenesis, we use “lipid homeostasis” to describe these lipid phenotypes as a whole. Importantly, we have not been able to uncouple the fat storage and vitellogenesis defects, which we view as a product of the overall impaired “lipid homeostasis”.

We have provided this clarification in the introduction (line 78): “Furthermore, impaired vitellogenesis can dramatically alter intestinal lipid levels, and conversely, metabolic dysfunction can downregulate vitellogenin production, with either event resulting in a defect in overall lipid homeostasis.”

Reviewer #2:

*The manuscript by Torzone et al reported signaling pathways that potentially worked in parallel (Follicle stimulating hormone-like) or downstream (p38 MAP kinase) of DRL-1, a protein that shares sequence similarity with mammalian mitogen-activated protein kinases. Based on results from forward genetic screens and targeted RNAi screens in the drl-1 mutant background, the authors proposed the convergence of cell-autonomous and non-cell-autonomous signals at the C. elegans intestine, which ultimately regulates the expression of a key lipoprotein gene vit-3. The same signaling network also appeared to regulate larval development and adult lifespan. Using CRISPR-based editing, this study created a large set of new transgenic lines that bore precise mutations and/or fluorescent protein/epitope tags. These lines allowed the authors to study proteins of interest without over-expression, which has the potential of clarifying a large body of work in a crowded field. However, to achieve this goal, the authors should demonstrate that the fluorescent protein/epitope tagged versions of DRL-1 and FLR-4 proteins retain full function. This is especially important because certain fluorescent proteins or epitope tags are known to perturb expression or function in specific contexts (*[*https://doi.org/10.1371/journal.pone.0183067*](https://doi.org/10.1371/journal.pone.0183067)*).*

Our *mKate2::3xFLAG::AID::drl-1* and *mNG::3xFLAG::AID::flr-4* CRISPR knock-in strains were used throughout the study to deplete DRL-1 or FLR-4 with auxin. In the absence of auxin, these strains are completely wild-type (see Fig 1B, 3A-B, 7D, 8C, S4B-F, etc.). Similarly, KIN-1::AID and FSHR-1::AID were wild-type in the absence of auxin. The *3xHA::flr-2* and *fshr-1::AID::3xFLAG* alleles were tested for their ability to suppress *drl-1* RNAi, which would indicate that they are loss-of-function; however, these strains were wild-type. Finally, the 3xHA::mGL::FLR-4 strain has wild-type growth rates.

*Major points:
1. [Line 3] "Follicle stimulating hormone signaling" in the title is slightly misleading because FSH does not exist in C. elegans. The authors may consider using "FSH-like signaling" instead.*

We agree with the Reviewer’s assertion that use of “follicle stimulating hormone signaling” may be misleading given the lack of clear conservation. Thus, we have changed the title to read “Glycoprotein hormone signaling…”. In the main text, we have now used “glycoprotein hormone signaling” or “follicle stimulating hormone-like” or “FSH-like” where appropriate.

*2. [Line 133] The use of col-10 promoter to drive transgene expression in the hypodermis may be flawed in this study. This is because col-10 is strongly expressed in larvae but not adults. Yet data reported in the manuscript (RNAi, and auxin-mediated protein degradation) were collected from day 1 adult animals. Although this may not affect the conclusion that DRL-1 acts in the intestine, the authors should consider alternative controls.*

We thank the Reviewer for their comments and for pointing out that the *col-10* promoter is poorly expressed at adulthood. We inspected the *reSi1[*P*col-10::TIR1::F2A::mTagBFP2::AID*::NLS::tbb-2 3'UTR]* strain for BFP expression in L4s and day 1 adults, finding clear BFP expression only in L4s (Fig S2A). However, it is likely that TIR1 protein remains in the hypodermis into adulthood because *reSi1[*P*col-10::TIR1]; kin-1::AID* animals placed on auxin as day 1 adults develop severe hypodermal defects within 24 hours (Fig S2B). Nonetheless, we performed a growth assay with auxin comparing *reSi1[*P*col-10::TIR1]; AID::drl-1* and *reSi5[*P*ges-1::TIR1]; AID::drl-1* animals at the L4 stage, finding that only intestinal depletion of DRL-1 impaired growth (Fig S2C), which is consistent with our previous findings.

*3. [Line 154] The definition of DRL-1 as a stand-alone MAP kinase is problematic. As stated by the authors, key residues for ATP binding and catalysis are missing in DRL-1. Although a prior publication (*[*https://doi.org/10.1111/acel.12218*](https://doi.org/10.1111/acel.12218)*) from another lab reported the ability of DRL-1 to phosphorylate a model substrate (Maltose Binding Protein), the results could be interpreted differently. Based on the method described, it was possible that proteins (including another kinase) that co-immunoprecipitated with DRL-1 from mammalian cells could be responsible for substrate phosphorylation. Therefore, the authors should consider an alternative model for DRL-1 action. Is it possible that DRL-1 is a non-catalytic partner of FLR-4? Is the temperature sensitive phenotype of the DRL-1(P269S) 'activation loop' mutant caused by structural instead of catalytic defect? Ideally, kinase assays would need to be performed with purified DRL-1 proteins, if the authors wish to draw a strong conclusion on its role as a kinase.*

We appreciate the Reviewer’s concerns regarding the kinase activity of DRL-1 and we have also considered that DRL-1 may be a pseudokinase. However, several points argue against this:

1) DRL-1 has in vitro kinase activity against myelin basic protein when immunoprecipitated from mammalian cells (Chamoli, Aging Cell 2014; Supplemental Figure 2). This paper is cited in the manuscript.
2) A single mutation in DRL-1, E123A, abrogates this in vitro kinase activity. This argues that a mammalian kinase is probably not being co-immunoprecipitated with DRL-1 from cells.
3) We find that the activation loop mutation *drl-1(P269S)* produces temperature-sensitive phenotypes in a manner that is identical to the *flr-4(P223S)* activation loop mutation. These mutations are positioned in a highly conserved Ala-Pro-Glu (APE) motif, which is required for the activity of v-Src.
4) Over-expression of DRL-1 suppressed the growth rate defects induced by simultaneous loss of both *drl-1* and *flr-4* (Fig S3D).

It is possible that the DRL-1(P269S) or FLR-4(P223S) mutations impact protein folding and not catalytic activity; however, the use of kinase assays would not be able to differentiate between these two possibilities.

We have softened our language in the text regarding the kinase activity of DRL-1, modifying “kinase dead” to “presumptive kinase dead” or “kinase activity” to “presumptive kinase activity”. Additionally, we provide this modification to the text (line 207): “Together, these data suggest that the kinase activity of DRL-1 and FLR-4 is required to maintain overall organismal homeostasis; however, we cannot rule out the possibility one or both proteins lack kinase activity and act as a non-catalytic partner to another protein kinase.”

*4. [Line 179] The diet-dependent lifespan extension of DRL-1 or FLR-4 deficient animals was difficult to follow. The authors should provide additional background information and spell out the significance of their results.*

We have modified our language to better explain the previously published result and how our data fits in this context. The text now reads (line 198): “Intriguingly, the FLR-4 P223S activation loop mutant exhibits a lifespan extension only when reared on *E. coli* HT115, and not OP50, suggesting that components in the diet interface with longevity pathways downstream of FLR-4 [20]. To investigate whether DRL-1 also restricts lifespan in a diet-dependent manner, we performed longitudinal lifespan assays on the *flr-4(P223S)* and *drl-1(P269S)* mutants fed different *E. coli* food sources. Indeed, the presumptive DRL-1 and FLR-4 kinase dead mutants are markedly long-lived on *E. coli* HT115 (Figs 2D and 2E). These findings argue that the kinase activity of both FLR-4 and DRL-1 are required to restrict longevity in response to diet.”

*5. [Line 205] The use of genetic mutant or RNAi to perturb the formation of lysosome related organelles (LROs) and reduce intestinal autofluorescence signals is an interesting approach, with caveats. The authors assumed that the block in LRO formation did not contribute to the localization of DLR-1 and FLR-1. Alternative imaging techniques for detecting weak fluorescence signals and subtracting autofluorescence should be attempted. In addition, co-localization of DRL-1 and FLR-4 cannot be used to address the statement in [Line 211]: "to test whether DRL-1 and FLR-4 form a protein complex".*

The *glo* mutants have been previously used to aid in imaging poorly expressed proteins, which we have adopted in our study to increase sensitivity of mKate2::DRL-1 and mGL::FLR-4 detection. We did attempt to image mKate2::DRL-1 in the absence of *glo-3* RNAi, but could not detect its expression above the gut autofluorescence. We have noted in the manuscript that our observations are consistent with previous over-expression studies: “we detected the mGL::FLR-4 and mKate2::DRL-1 proteins at the intestinal plasma membrane (Fig 3D), consistent with over-expression studies of FLR-4 and DRL-1 [29,30]”. Importantly, we also performed our co-IP studies in the *glo-4* mutant background and found that FLR-4 and DRL-1 form a protein complex (consistent with our genetic data), suggesting that the *glo-4* mutation does not negatively impact FLR-4/DRL-1 localization or binding.

We have modified the text from "To test whether DRL-1 and FLR-4 form a protein complex" to “To test whether DRL-1 and FLR-4 co-localize”, which is more appropriate (line 263).

*6. [Line 215] To properly assess the efficiency of immunoprecipitation (enrichment factor), the authors should state the amount of whole cell lysate loaded in relation to the amount of lysate used for immunoprecipitation, i.e. % input. Based on Fig. 3E, lower panel, FLR-4 was less efficient than DRL-1 in pulling down DRL-1. The authors should elaborate on the significance of this result.*

We have added updated the figure legend to include the amount of whole cell lysate loaded relative to that used for the IP.

It is difficult to compare between the two reciprocal co-IPs since different antibodies were used for 1) the IP component and 2) the blotting component of the experiment. In our hands, the 3F10 anti-HA antibody is highly sensitive, with low background signal, when used in a Western blot, while the M2 anti-FLAG antibody lacks this sensitivity. It is also difficult to assess IP efficiency between the antibodies. Thus, we can’t comment on the biological significance of the difference in signal between the two reciprocal co-IPs.

*7. [Line 390] Similar to point 4, the use of glo-3 RNAi to reduce auto-fluorescence for TIR-1::wrmScarlet imaging is not free of caveats. Separately, although it is not the authors' burden to demonstrate that TIR-1::wrmScarlet puncta formation equates phase transition, please consider toning down [Line 392] "loss of drl-1 induces TIR-1::wrmScarlet phase transition". There is a similar problem with [Lines 36-37]. Have TIR-1 puncta been detected when TIR-1 was fused to different fluorescent protein partners?*

The *glo-3* RNAi has been previously used to aid in the imaging of TIR-1::wrmScarlet (Peterson, eLife 2022), which we adopted during our study to increase sensitivity. Nonetheless, we have also performed TIR-1::wrmScarlet imaging in absence of *glo-3* RNAi, finding that loss of *drl-1* again stimulates puncta formation. This analysis is shown below, although we are not including this in the manuscript since this *glo-3* method has already been established.

While we acknowledge that we have only demonstrated oligomerization of TIR-1 (via cell biological approaches) and not phase transition, it has been established that TIR-1 undergoes phase transition to activate its NAD+ glycohydrolase activity using rigorous biochemical approaches (Peterson, eLife 2022). Because this previous work has demonstrated that TIR-1 puncta formation in worms corresponds to TIR-1 phase transition *in vitro*, we believe that the use of “phase transition” is appropriate in our study. Nonetheless, we have modified this text to read “suggesting that loss of *drl-1* likely induces TIR-1::wrmScarlet phase transition” (line 482) and “signaling through DRL-1 and FLR-2 coordinates TIR-1 oligomerization” (lines 40-41). Additionally, we have replaced “phase transition” with “oligomerization” in several locations within the manuscript.

TIR-1::wrmScarlet phase transition is induced specifically upon loss of *drl-1*, which is partially suppressed in the *drl-1; flr-2* double mutant. Loss of *flr-2* alone or hyperactivation of KIN-1 (*i.e.*, the *kin-2* mutant) does not stimulate TIR-1::wrmScarlet phase transition. Together, these data argue that phase transition is a specific response to loss of *drl-1* and not an artifact of the tag.

*8. [Line 402] Since pha-4 RNAi could regulate Pvit-3::mCherry reporter activity in drl-1 mutant, are PHA-4 binding sites found in the vit-3 promoter? The images in Fig. 8C and Fig. S9A were hard to follow. Although the method for measuring PHA-4::GFP nuclear signal was described in the Materials and methods section, readers who are unfamiliar with C. elegans anatomy would be confused by the images at such low magnification. The addition of magnified insets with nuclei marked should be considered.*

We inspected the published PHA-4 ChIP-Seq data generated by the modENCODE project. PHA-4 binds to the promoter of the *vit-3* and *vit-4* genes at moderate levels when compared to promoters that are strongly bound by PHA-4 (Fig. S12). Notably, binding to the *vit* promotes is specific to adulthood, which is consistent with vitellogenin expression. It is possible that PHA-4 is driven to the nucleus at adulthood due to basal levels of PMK-1 activity.

To describe this finding, we have added the following text: "It is possible that PMK-1 promotes the nuclear accumulation of PHA-4, where it directly represses the expression of specific developmental genes. In support of this hypothesis, PHA-4 is found at the promoters of the *vit* genes at low levels specifically at adulthood (S12C Fig), possibly mediated by the basal activity of PMK-1.” (line 536).

We have now included blowup images of PHA-4::GFP nuclear localization in Fig 8C and Fig S9A.

Reviewer #3:

*Torzone et al. uses C. elegans to identify the two opposing, inhibitory and stimulatory, effects on the p38 MAPK pathway that regulates metabolic homeostasis during development. They show that mutations in the kinases, DRL-1 and FLR-4 that seem to form a complex on the intestinal membrane, engage the p38 MAPK to prevent vitellogenesis and reduce growth. In a forward genetic screen, they identify the FLR-2 (neuronal)/FRSH-1 (intestinal) axis as the neurohormonal signaling pathway that stimulates the p38 MAPK through a G-protein signaling, leading to phase transition of p38 MAPK upstream modulator, TIR-1. Together, the authors uncover an intricate signaling network that regulates organismal growth and development. However, it should be noted that both FLR-4 and DRL-1 do not have direct orthologs in other systems and may be restricted to a nematode sub-clade. So, the global appeal will reduce if the context of the study is not well defined. It is not clear under what circumstances the opposing pathways work; what are the individual triggers for the two arms.

While a part of the work is quite interesting, the study seems premature in multiple aspects:

Major comments:
1) This reviewer is not convinced by the model that the study has proposed. Is it not possible that flr-2 works downstream of drl-1/flr-4 that negatively regulates it? The alternative possibilities should be tested and eliminated.*

We thank the Reviewer for their suggestions and agree that it is possible for *flr-2* to function genetically downstream of *drl-1/flr-4*. As pointed out, this model would require that DLR-1/FLR-4 negatively regulate FLR-2, which in turn would restrict development and activate p38 signaling. To test this hypothesis, we inserted a HA tag into the *flr-2* locus by CRISPR/Cas9 editing (following the secretion signal cleave site), performed *drl-1* or *flr-4* RNAi, and determined FLR-2 levels by Western blot analysis. Loss of *drl-1* or *flr-4* did not increase FLR-2 levels (Fig S7B). Moreover, constitutive over-expression of *flr-2* in the *drl-l* mutant did not further impair development (Fig S7C). Finally, loss of *flr-2* alone did not impact TIR-1 oligomerization (Fig. S11B-C). Together, these data argue that DLR-1/FLR-4 do not negatively regulate FLR-2 and that the FLR-2 pathway acts in parallel to MAPK signaling.

We have added the following text (line 326) “Notably, knockdown of *drl-1* or *flr-4* did not alter FLR-2 levels, and constitutive over-expression of *flr-2* in neurons did not dramatically suppress or enhance the body size defects exhibited by the *drl-1* mutant, which together, indicate that *flr-2* acts in parallel, and not downstream, of *drl-1* (S7B-C Figs).”

*2) What are the conditions that will activate flr-2/fshr-1? The authors should show what happens to TIR-1 phase transition when they activate that arm independently.*

We have focused our study on the developmental function of *flr-2/fshr-1* during impaired MAPK signaling. While the role of this pathway in other conditions or physiological contexts is clearly important, including understanding whether FLR-2 functions in stress responses or during aging, this work is outside of the scope of our developmental study.

We thank the Reviewer for their suggesting to test TIR-1 oligomerization in response to loss or hyperactivation of the FLR-2/FSHR-1/PKA pathway without perturbing DRL-1/FLR-4 function. Surprisingly, we found that loss of *flr-2* or *kin-2* (which activates PKA) has no effect on TIR-1 oligomerization (Fig S11B-C). Moreover, activating mutations in PKA signaling do not induce phosphorylation of PMK-1 (Fig S11D). These data argue that PKA signaling does not act directly on TIR-1 and we have changed our model to indicate that FLR-2 acts through a PKA-independent pathway to modulate TIR-1 phase transition (Fig 8E).

We have described this result in the main text and changed our language regarding the interaction between PKA and p38 signaling (line 506): “Our results suggest that FLR-2/FSHR-1 signaling stimulates p38 activity in the absence of *drl-1*/*flr-4*, however it remains unclear whether PKA mediates these effects downstream of FLR-2. A gain-of-function mutation in Gα_s_, *gsa-1(ce81),* or a loss-of-function mutation in the inhibitory regulator of PKA, *kin-2(ce179),* hyperactivates PKA signaling [48] and reduces body size. This body size defect is partially suppressed by *pmk-1* RNAi (S11A Fig), suggesting that PKA functions either upstream or in parallel to PMK-1. To directly test whether PKA activation stimulates p38, we measured TIR-1 oligomerization and PMK-1 phosphorylation in the PKA hyperactivation mutants, finding that independent activation of PKA failed to induce TIR-1 phase transition or PMK-1 phosphorylation (S11B-D Figs). While it is possible PKA only acts on the p38 pathway when *drl-1* or *flr-4* is lost, it is more likely that FLR-2 stimulates a PKA-independent parallel pathway, possibly mediated by an alternative G protein signaling cascade, to modulate the activity of TIR-1.”

*3) The authors fail to establish the flr-2/fshr-1 axis. While they are independently shown to modulate p38 MAPK, there is no data suggesting that they are in the same pathway. Same is the case with the G protein signaling.*

Because the *flr-2* and *fshr-1* mutations suppress the *drl-1*/*flr-4* mutations to similar levels and because glycoprotein hormones (FSH, LH, and TSH) are known to act through Gα_s_/AC/PKA signaling in mammals, it is likely that FLR-2, FSHR-1, PKA all act in the same pathway. These points are made in the manuscript. However, it is possible that FSHR-1 may couple to other G proteins to activate other signaling cascades, which is beyond the scope of this study.

In support of the conclusions from our genetic studies, a recent publication demonstrated that FLR-2 and FSHR-1 biochemically interact. We have added this citation to the manuscript and the following text (line 401): “Indeed, FLR-2 was recently shown to bind to the extracellular domain of FSHR-1 in response to freeze-thaw stress [39], further supporting our conclusion that FLR-2 and FSHR-1 act in the same pathway.”

*4) When showing neuron-specific flr-2 depletion, they need to show intestine-specific depletion as a control. Vice versa for fshr-1. Same for tissue-specific RNAi for the G-protein signaling.*

To examine the tissue-specificity of *flr-2*, we performed pan-neuronal rescue of *flr-2* in the *flr-2* mutant, which was successful and is consistent with previous studies showing *flr-2* is exclusively expressed in neurons. Because FLR-2 is a secreted hormone, it is possible that *flr-2* rescue in other tissues may yield a similar phenotype. Thus, we inserted an AID tag into the *fshr-1* locus to perform tissue-specific depletion experiments using auxin to supplement our tissue-specific RNAi results; however, we were unable to effectively deplete FSHR-1::AID with auxin (based on phenotypes), possibly because it is a membrane protein or because the C-terminus is not accessible to the degradation machinery. Finally, we inserted two AID tags into the *kin-1* locus, which labeled all isoforms, and performed tissue-specific depletion of KIN-1 (Fig 6E-G). These results were consistent with our *fshr-1* tissue-specific RNAi experiments and argue that FSHR-1/GSA-1/ACY-4/KIN-1 signaling occurs in the intestine.

The new text is as follows (line 389): “Notably, knockdown of *kin-1* by RNAi failed to strongly suppress the vitellogenesis and body size defects exhibited by the *drl-1* mutant. To investigate the role of KIN-1 in this pathway more rigorously, we introduced AID tags into two locations within the *kin-1* locus using CRISPR/Cas9 editing, resulting in AID-tagging of all the *kin-1* isoforms. Depletion of KIN-1 in the intestine, but not in neurons, suppressed the vitellogenesis and body size defects resulting from *drl-1* knockdown (Figs 6E and 6F), which is consistent with our intestine-specific RNAi experiments. Interestingly, hypodermal depletion of KIN-1 at the L4 stage results in a highly penetrant bursting phenotype (Fig 6G), indicating that *kin-1* is required for developmental programs in the hypodermis or seam cells.”

*5) The authors should confirm that the kinase dead version of the proteins that they have generated are indeed biochemically deficient in kinase activity.*

The DRL-1 E253A mutation has been shown to severely disrupt kinase activity *in vitro* (Chamoli, Aging Cell 2014; Supplemental Figure 2). Moreover, the activation loop mutations, *drl-1(P269S E270K)* and *flr-4(P223S)*, fall in a highly conserved Ala-Pro-Glu (APE) motif, which is required for the activity of v-Src. The APE motif is completely conserved in FLR-4, suggesting that the *flr-4(P223S)* mutation would disrupt kinase activity (like v-Src). Our analysis of DRL-1 used the *drl-1(E253A G254A P269S E270K)* mutant, which lacks resides required for *in vitro* kinase activity and contains the activation loop mutations. While these mutant proteins are likely to lack kinase activity, we have modified our language in the text from “kinase dead” to “presumptive kinase dead” or “kinase activity” to “presumptive kinase activity”.

*6) The authors should show kinase activity when they refer to the hyperactivation of the p38 MAPK.*

We have demonstrated p38 hyperactivation using two independent methods: 1) imaging and quantification of TIR-1 oligomerization using cell biological approaches (Figs 7F-I and S11B-C) and 2) Western blotting against phospho-PMK-1 (Figs S9 and S11D). These analyses provide a measure of tissue-specific p38 activation (TIR-1 imaging) and whole animal PMK-1 activity (Western blotting), which meets the standard in the field. The use of p38 “hyperactivation” has been widely used in the literature to describe similar phenotypes (for example, see Cheesman, G3 2016 or Weaver, Dev Cell 2020), thus we believe that this language is justified in our study.

*7) The authors claim that the two proteins form a complex on the membrane using colocalization and co-IP. While the co-IP data is convincing, the colocalization is not. The nature of the complex is also not studied. Will overexpression/activation of one protein suppress the other?*

As discussed above, the mKate2::DRL-1 and mGL::FLR-4 proteins are exceptionally difficult to detect in the intestine. Nonetheless, our observation that DRL-1 and FLR-4 are both found at the plasma membrane are consistent with over-expression studies of FLR-4::GFP (Kobayashi, Genes to Cells 2011) and DRL-1::GFP (Wimberly, Genetics 2022), as cited in our manuscript. Our reciprocal co-IP data demonstrate that the proteins interact biochemically and is consistent with the notion that they co-localize in cells.

The concept that over-expression of one protein may suppress loss of another is intriguing. Thus, we chose to over-express DRL-1 in the *drl-1(rhd109)* mutant while simultaneously knocking-down *flr-4* by RNAi. Over expression of DRL-1 suppressed the growth rate defects upon simultaneous loss of both *drl-1* and *flr-4* (Fig S3D). We have included the following text in the manuscript (line 205): “Consistently, over-expression of *drl-1* on an extrachromosomal array is sufficient to suppress the growth rate defects induced by simultaneous loss of both *drl-1* and *flr-4* (S3D Fig), suggesting that DRL-1, and its potential kinase activity, can compensate for loss of *flr-4*.”

*8) Line 237: does the drl-1;flr-2 suppress the increased life span of drl-1?*

The *flr-2* mutation fails to suppress the lifespan extension conferred by loss of *drl-1* when animals are reared on *E. coli* HT115 (Fig 4H). We have discussed this finding in the paragraph beginning on line 331. We don’t believe any further discussion is warranted.

*9) Line 348: The authors get only partial suppression of phenotype. Have they checked sek-1 or nsy-1 mutants? Is it possible that other PMKs are required?*

We agree that it is possible that other PMKs may be required for *drl-1/flr-4* mutant phenotypes. Thus, we repeated the body size analysis shown in Figure 7D and included the *tir-1(qd4)* null mutant. Loss of *tir-1* suppressed DRL-1 depletion to a similar degree as the *pmk-1* null mutant, suggesting that other PMKs are not involved.

*10) The phase transition of TIR-1 is not unexpected. They should have used the oligomerization defective or enzymatic action defective versions as control.*

The phase transition of TIR-1 was recently shown to be required for PMK-1 activation upon pathogen infection or during abiotic stress responses (i.e., cholesterol deficiency), which is dependent on key residues in the TIR domain (Peterson, eLife 2022). Those residues have already been characterized in that study. In addition to TIR-1 phase transition, we have provided genetic and biochemical evidence of PMK-1 activation following loss of *drl-1/flr-4* in our study. We do not believe that inclusion of these new experimental controls would alter the conclusions already presented in the manuscript.

*11) The PHA-4 data is incomplete without proper understanding of what genes the transcription factor regulates in this context. How is the transcription factor activated?*

I agree with the Reviewer’s assertion that understanding the mechanism of PHA-4 activation in *drl-1* mutants, as well as identifying the transcriptional targets of PHA-4 in this context, is critically important; however, this work represents a major undertaking to gain this level of mechanistic insight. We believe that this work is beyond the scope of this manuscript and is appropriate for a follow up study.

*12) The authors have observed severe developmental defects in flr-4(n2259) worms. This contradicts the study from Verma et al, 2018 and Nair et al, 2021. Authors should comment on this.*

We appreciate the Reviewer’s comments regarding the inconsistencies between the developmental phenotypes that we observed in *flr-4(n2259)* mutants and those described by Verma et al. 2018. Firstly, the Nair et al. 2021 study examined *flr-4(n2259)* developmental phenotypes in the context of propionate supplementation, which we did not test in our study. We show that *flr-4(n2259)* mutants display only modest body size and vitellogenesis defects when reared at 20°C (Fig 2C and Fig S3A). These data are consistent with the data shown in Verma et al 2018, displayed in Supplemental Figure 2D, where *flr-4(n2259)* mutants reared at 20°C display no significant body size defects. Notably, the data displayed in Supplemental Figure 3 of the Verma paper shows that *flr-4(n2259)* do exhibit a modestly slowed developmental rate at 20°C (at 60hr wild-type animals were L4/young adults, *flr-4(n2259)* were L3/L4s); however, this phenotype was not discussed in that paper and we did not test *flr-4(n2259)* developmental rates. Together, our data and Verma’s data argue that *flr-4(n2259)* is a weak hypomorphic allele at 20C.

At 25C, we find that the developmental defects are stronger, a temperature that was never tested in Verma et al, 2018. However, in Take-uchi et al 2005, the authors describe that “two *flr-4* mutants, *n2259* and *sa201*, which showed temperature-dependent abnormality in defecation cycle periods, also showed temperature-dependent resistance to 400 μg/ml NaF; namely, they were resistant to NaF at 25°C but sensitive at 20°C.” The Take-uchi data are completely consistent with our findings.

Therefore, we believe that our description of the *flr-4(n2259)* allele is consistent with all previously published data. To clarify our findings, we have modified our language in the text and have cited the Take-uchi paper (line 165): “To address whether DRL-1 possesses kinase activity we used CRISPR/Cas9 to generate a DRL-1 P269S mutation, which is analogous to the FLR-4 P223S hypomorphic mutation that is positioned within the activation loop of the kinase domain and yields temperature-sensitive phenotypes [19].”

*13) In line 180, the statement that flr-4 RNAi increases life span only on HT115, but not OP50 is incorrect. In that paper (Verma et al 2018), the authors show that life span is extended on both HT115 and OP50 flr-4 RNAi; this was not the case with the n2259 allele.*

We appreciate the reviewer bringing this point to our attention. Verma observed that *flr-4* RNAi extended the lifespan of wild-type animals when the RNAi was delivered in HT115 or OP50, as seen in Figure 1D of their paper. They also observed diet-dependent lifespan extension of *flr-4(n2259)* mutants when animals are specifically consuming an *E. coli* HT115 diet, which is consistent with the data we show in Figure 1D and 1E in this manuscript. We have adjusted the language in our manuscript correct this error (line 198): “Intriguingly, the FLR-4 P223S activation loop mutant exhibits a lifespan extension only when reared on *E. coli* HT115, and not OP50, suggesting that components in the diet interface with longevity pathways downstream of FLR-4 [20]”
