# Supplementary material for: Opposing action of the FLR-2 glycoprotein hormone and DRL-1/FLR-4 MAP kinases balance p38-mediated growth and lipid homeostasis in C. elegans
Source: PLoS Biol. 2023 Sep 29;21(9):e3002320. doi: 10.1371/journal.pbio.3002320 (PMC10566725; doi:10.1371/journal.pbio.3002320)
Supplement: Attachment — Submitted filename: Response_to_Reviews.docx [file pbio.3002320.s042.docx]

***Response to editors and reviewers’ comments:***

*Editorial requests:*

*1) TITLE: After some discussion within the team, we think the title could be streamlined a bit. Therefore, if you agree, we suggest you change the title to "Opposing action of glycoprotein hormone and DRL-1/FLR-4 MAP kinases balance p38-mediated growth and lipid homeostasis in C. elegans"*

We have modified the title to “Opposing action of the FLR-2 glycoprotein hormone and DRL-1/FLR-4 MAP kinases balance p38-mediated growth and lipid homeostasis in *C. elegans*”. We are concerned that the title proposed above may be confusing, as there are several glycoprotein hormones encoded in the *C. elegans* genome. Because of this, we would prefer our updated title or our original title.

*2) BLURB: In the relevant portion of our online system, please provide a blurb which (if accepted) will be included in our weekly and monthly Electronic Table of Contents, sent out to readers of PLOS Biology, and may be used to promote your article in social media. The blurb should be about 30-40 words long and is subject to editorial changes. It should, without exaggeration, entice people to read your manuscript. It should not be redundant with the title and should not contain acronyms or abbreviations.*

We have submitted the blurb during the online submission.

*3) BLOTS AND GELS: Please note that we require the original, uncropped and minimally adjusted images supporting all blot and gel results reported in an article's figures or Supporting Information files. We will require these files before a manuscript can be accepted so please prepare and upload them now. Please carefully read our guidelines for how to prepare and upload this data:* [*https://journals.plos.org/plosbiology/s/figures#loc-blot-and-gel-reporting-requirements*](https://journals.plos.org/plosbiology/s/figures#loc-blot-and-gel-reporting-requirements)*. Please provide the uncropped and minimally adjusted images relating to Fig 3E; Fig S7A-B; Fig S9A-B; Fig S11D*

The minimally adjusted images relating to these figures are now provided in raw_images.pdf files S1-S6, which have been submitted as Supporting Information files. We have referenced these files in the appropriate figure legends.

*4) DATA: You may be aware of the PLOS Data Policy, which requires that all data be made available without restriction:* [*http://journals.plos.org/plosbiology/s/data-availability*](http://journals.plos.org/plosbiology/s/data-availability)*. For more information, please also see this editorial:* [*http://dx.doi.org/10.1371/journal.pbio.1001797*](http://dx.doi.org/10.1371/journal.pbio.1001797)*. Regardless of the method selected, please ensure that you provide the individual numerical values that underlie the summary data displayed in the following figure panels as they are essential for readers to assess your analysis and to reproduce it: Fig 1C-E; Fig2B-E; Fig 3B-C; Fig 4B-H; Fig 5B-C,E; Fig 6C-F; Fig 7B-E,G-I; Fig 8B,D, Fig S1D-E; Fig S2B-C; Fig S3B-D; Fig S4D-F; Fig S5A-C; Fig S6C-D; Fig S7C; Fig S8B; Fig S10B-C; Fig S11A-C; Fig 12B*

The raw data underlying the summary data presented in these figures are now provided in Data.xlsx files S1-S19, which have been submitted as Supporting Information files.

*NOTE: the numerical data provided should include all replicates AND the way in which the plotted mean and errors were derived (it should not present only the mean/average values).*

We have included all replicate measurements and provided the summary calculations that are presented in the figures.

*Please also ensure that figure legends in your manuscript include information on where the underlying data can be found, and ensure your supplemental data file/s has a legend.*

We have added this information to each figure legend.

*Please ensure that your Data Statement in the submission system accurately describes where your data can be found.*

This has been updated to include our Data.xlsx and raw_images.pdf files.

Reviewer #2: *Minor comments:
1. Please add arrowheads to specify nuclear GFP signals in Fig. 8C and S12A.* 

We have added arrowheads pointing to the intestinal nuclei shown in the magnified images in Fig. 8C and S12A.

*2. [Line 573] All *stains* used in this study are listed in S1 File.   Please correct typo.* 

This has been corrected.

Reviewer #3:

*The phase transition images are still not convincing to this reviewer and authors should consider placing high-resolution images as a supplementary figure. In the publication from Pukkila-Worley group in Elife that reported the phase transition, the number of puncta increases whereas here it does not, and only the size increases. How this may be reconciled? So, the use of the controls that were suggested in the first round of review (reviewer 3, point 10) is important to perform.* 

We thank the reviewer for their comments. The representative images in Fig 7F are high-resolution images that have been denoised and deconvoluted to produce the best contrast between the TIR-1 puncta and the background autofluorescence, as described in the methods. To better demonstrate the puncta, we have now included magnified images in Fig 7F for all three TIR-1 samples and have updated the figure legend to reflect this change.

Based on personal communications with the Pukkila-Worley group, we believe that the difference in our findings is due to imaging and analysis approaches. Alternatively, *drl-1* may impact TIR-1 oligomerization differently than pathogen or cholesterol stress (as reported by Pukkila-Worley group in Elife). We have added these two possibilities to our discussion (lines 411-417):

“Notably, previous work has demonstrated that activation of TIR-1 by pathogen or nutrient stress stimulates an increase in TIR-1 puncta number [24]; however, we observed a modest decrease in the number of TIR-1 puncta and a dramatic increase in puncta size. It is possible that our imaging and analysis approach, using a different microscopy setup and deconvolution/denoising software, accounts for this difference. Alternatively, loss of *drl-1* may impact TIR-1 oligomerization differently than pathogen or nutrient stress. Nonetheless, we find that robust TIR-1 oligomerization correlates with activation p38/PMK-1 signaling, which is consistent with the previous findings [24].”

*In the Figure 6B and C, kin-1 RNAi does not reactivate Pvit-3::mcherry in the drl-1 mutant. However, the kin-1 can increase the body size. Since it is the terminal molecule of the pathway that authors propose to communicate to TIR-1, how is this point reconciled?* 

We thank the reviewer for pointing this out. We believe that this is a technical aspect of the *kin-1* RNAi clone that we chose to use in the experiment. Initially we tested four different RNAi clones to achieve knockdown of *kin-1* and all but one resulted in L1 arrest or severe developmental phenotypes, which made assessment of the vitellogenesis phenotype impossible. Thus, we chose the weakest *kin-1* RNAi clone to measure body size and fluorescent reporter expression of the *Pvit-3::mCherry* reporter in day one adult animals. It is likely that this clone achieved only partial knockdown of *kin-1* transcripts, as it did not result in lethality and it did not, as the reviewer points out, strongly reactivate *Pvit-3::mCherry* expression. We have added more information regarding the *kin-1* RNAi clones to the methods section (lines 523-526): “We selected four different *kin-1* RNAi clones from the Ahringer library to initially test for their ability to suppress the *drl-1* mutant phenotypes. Three of these RNAi clones caused lethality or severe developmental phenotypes, while the weakest clone permitted growth to adulthood but produced modest *drl-1* suppression phenotypes, likely due to incomplete knockdown of the *kin-1* transcript.”

To further investigate the role of KIN-1/PKA as the terminal molecule of this pathway, we introduced 2 AID tags into the *kin-1* locus to apply temporal degradation of KIN-1 to avoid the arrest/lethality that we observed with strong *kin-1* RNAi. Degradation of KIN-1 in the intestine by the auxin-inducible degron system does strongly reactivate *Pvit-3::mCherry* expression and increase body size in *drl-1* mutants (Figs 6E-F). We have also performed this analysis after depletion of KIN-1 in neurons (Fig 6E-F) and hypodermis (Fig 6G). All of these data and the supporting text was included in the last version of the manuscript (see lines 280-288), and we don’t believe that additional changes are warranted. Finally, we had changed our model in Fig. 8E during the previous submission to not include direct communication between KIN-1 and TIR-1 based on data shown in Fig S11B-D. This change was described in detail in the “response to reviews” document in the second submission.

*In figure S8A, line 344, the representative image of nsy-1, sek-1 doesn't show reactivation of Pvit-3::mcherry in the drl-1 mutant while the quantification shows that. Why is it so?*

We thank the reviewer for their inquiry. We assume that they are referring to Fig S10A, and not S8A, given their comment. It is likely that the *nsy-1* and *sek-1* RNAi clones used in this study are weak and achieve only partial knockdown of their target transcripts, as has been described previously. The quantification plot that the reviewer is referring to is Figure S10B and is a set of body size measurements and not *Pvit-3::mCherry* reporter quantification. We hypothesize that more complete knockdown of *nsy-1*/*sek-1* transcripts, or use of genetic mutants, would more strongly reactivate the *Pvit-3::mCherry* reporter in *drl-1* mutants, as we reported with the *tir-1* and *pmk-1* mutants (Fig 7D). Thus, we don’t believe that modifications to the existing text are warranted.

*It is not clearly mentioned how many times the western blot experiments of Fig S7 were performed. Same with the IP experiment in Fig 3E. It will be great if the authors present all the replicates in the source data file for the readers to understand the variability they may expect.*

All Western blots were performed at least twice with similar results. We have added this language to the legends for each figure that contains Western blot data. We have added the source data in raw_images.pdf files S1-S6 for the Western blot data presented in each figure.

*In the figure 3E, is a band expected in the WCL of FLAG western? If yes, why is it not visible here?* 

The CRISPR-tagged HA::FLR-4 and FLAG::DRL-1 proteins are expressed at endogenous levels, which we found to be difficult to detect by Western Blot. Although we loaded equal amounts of protein for each blot, only HA::FLR-4 could be detected in the whole cell lysate sample. This was reproducible across experiments. To account for this observation, we have added “FLAG::DRL-1 was not detectable in the WCL likely due to low levels of protein expression or poor sensitivity of the anti-FLAG antibody.” to figure legend on line 920 in the text.

*I could not find the source data file for the experiments. If it is not attached to the revised document, the authors may consider putting all primary data in a source file.*

We have now provided all the source data in Data.xlsx files S1-S19 and raw_images.pdf files S1-S6.

*In the legend, it will be great if the authors clearly mention the number of replicates (technical and biological) used for each experiment.*

The replicate data, including numbers, can now all be found in the Data.xlsx files S1-S19.
